# Hydrogeologic Drivers and Management Implications of PFAS, Nitrate, and Pharmaceuticals in Groundwater at a Karst Beneficial Reuse System

**DOI:** 10.1021/acsestwater.6c00547

**Published:** 2026-07-28

**Authors:** Kelly Kosiarski, Tamie L. Veith, Faith A. Kibuye, Patrick J. Drohan, Onur G. Apul, Heather E. Preisendanz

**Affiliations:** † Department of Agricultural and Biological Engineering, 8082 The Pennsylvania State University, University Park, Pennsylvania 16802, United States; ‡ USDA-Agricultural Research Service, Pasture Systems and Watershed Management Research Unit, University Park, Pennsylvania 16802, United States; § Department of Ecosystem Science and Management, 8082 The Pennsylvania State University, University Park, Pennsylvania 16802, United States; ∥ Penn State Extension, The Pennsylvania State University, University Park, Pennsylvania 16802, United States; ⊥ Department of Civil and Environmental Engineering, 6251 The Pennsylvania State University, University Park, Pennsylvania 16802, United States; # Institute of Sustainable, Agricultural, Food and Environmental Science, College of Agricultural Sciences, The Pennsylvania State University, University Park, Pennsylvania 16802, United States

**Keywords:** aquifer vulnerability, contaminants of emerging concern, DRASTIC model, fate and transport, recharge dynamics, wastewater reuse

## Abstract

Management of beneficial reuse sites typically focuses on controlling the fate and transport of conventional water quality parameters, particularly nitrogen. However, it is unclear if current management is also effective for controlling contaminants with distinct physiochemical properties, such as pharmaceuticals and per- and polyfluoroalkyl substances (PFAS). We investigated the spatial and temporal patterns of nitrate, PFAS, and pharmaceuticals in groundwater monitoring wells at a beneficial reuse site. These data sets were integrated with hydrogeologic variables, including groundwater elevation, rainfall, surface karst features, wastewater inputs, and irrigation intensity, to compare drivers of occurrence, fate, and transport behavior across classes of contaminants. Additionally, we evaluated the suitability of a modified version of a groundwater vulnerability model for predicting spatial patterns of PFAS and pharmaceuticals. Overall, pharmaceuticals exhibited lower detection frequencies, higher temporal variability, and spatial patterns distinct from nitrate and PFAS. Their episodic occurrence responded strongly to wastewater inputs and rarely exceeded criteria for antibiotic resistance. In contrast, PFAS and nitrate concentrations fluctuated primarily with groundwater elevation and were well-aligned with groundwater vulnerability predictions. Results suggest that reuse management should target periods of high rainfall and consumer use to reduce pharmaceutical inputs, whereas nitrate and PFAS management should be informed by groundwater conditions.

## Graphical abstract

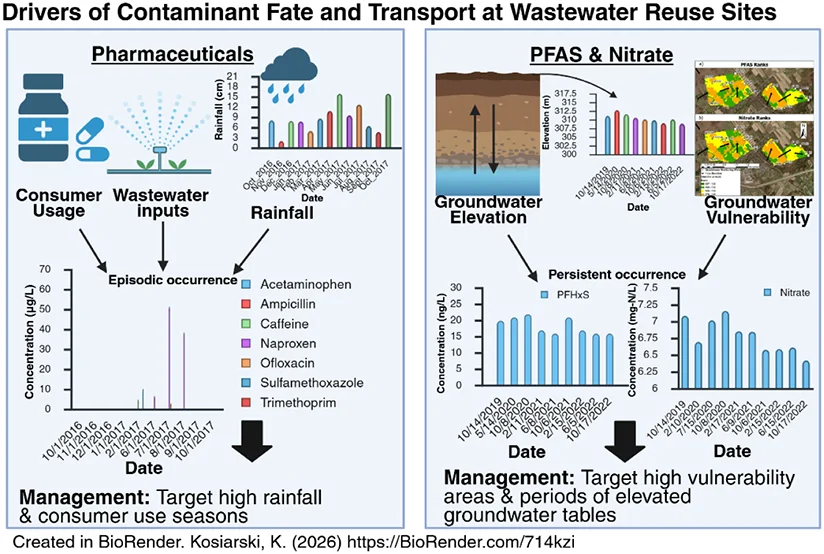

## 1 Introduction

Beneficial reuse of effluent from treated wastewater is generally considered to be a sustainable practice that reduces reliance on freshwater supplies and synthetic fertilizers.^1^ With 40% of the global population living in water-stressed regions,^2^ there is an increased need for sustainable water resource management.^3^ However, regulations for wastewater irrigation vary widely, and many countries still use untreated wastewater for irrigation.^3,4^ In the United States, federal regulations regarding the beneficial reuse of treated effluent primarily focus on conventional water quality parameters such as nutrients, pathogens, and heavy metals.^5,6^ While this regulatory approach has been effective for addressing eutrophication and other traditional human health risks, its effectiveness in addressing additional organic contaminants with distinct physiochemical properties, such as pharmaceuticals and per- and polyfluoroalkyl substances (PFAS), is unclear.

Pharmaceuticals and PFAS have attracted increasing attention from scientists, policymakers, practitioners, and the general public.^7,8^ Because they are not easily removed with current wastewater treatments and have a strong environmental persistence, growing evidence suggests that these compounds may pose risks to ecosystem and human health in beneficial reuse systems.^9^ Groundwater concentrations of pharmaceuticals and PFAS at other beneficial wastewater reuse sites are well documented and range from 10^–1^ to 10^2^ μg/L for pharmaceuticals^10^ and 10^–2^ to 10^2^ ng/L for PFAS.^11–13^

The transport and persistence of pharmaceuticals and PFAS inadvertently introduced to beneficial reuse practices are driven by site-specific factors such as soil properties, aquifer media, depth to groundwater, recharge profile, land use, and application rate and history.^10,11,14,15^ However, how these groups of chemicals behave spatially and temporally relative to one another and alongside conventional nutrients like nitrate remains unclear. The DRASTIC model assesses intrinsic groundwater vulnerability by integrating local hydrogeologic parameters to evaluate relative susceptibility.^16^ It provides a potentially useful tool for examining how hydrogeologic variability may influence the occurrence and transport of emerging contaminants in groundwater at beneficial reuse sites; however, it has been most widely applied to assess groundwater vulnerability to nitrate.

The overarching goal of this study was to address critical gaps in understanding how emerging contaminants (PFAS and pharmaceuticals) behave relative to a conventional water quality parameter (nitrate) at a spray-irrigation beneficial reuse system. This study extends the temporal scale of previous data collected at this site.^17^ Furthermore, it introduces an explicit investigation of the underlying transport factors across multiple contaminant classes (PFAS, pharmaceuticals, and nitrate) to identify shared hydrogeologic drivers in order to assess implications for monitoring and management strategies.

## 2 Methodology

### 2.1 Study Site

The study site is a 245-ha beneficial reuse site located in Central Pennsylvania. Treated wastewater effluent has been spray-irrigated since 1963;^18^ however, full-scale operations began in 1982. Wastewater source and treatment are summarized in Kibuye et al.,^19^ with additional details provided in Supplemental Text S1. The site is underlain by carbonate karst geology and is a mix of fracture-dominated and conduit-dominated karst.^20^ Surface karst features are shown in Figure 1. Land use at the site is half forested and half cropland. Synthetic fertilizer rates vary with average annual applications of 83 kg-N ha^–1^ for wheat, 120–220 kg-N ha^–1^ for cool season grasses, and 200 kg-N ha^–1^ for corn. Pesticide applications occur only on cultivated cropland at the site (Figure 1).

**Figure 1:**
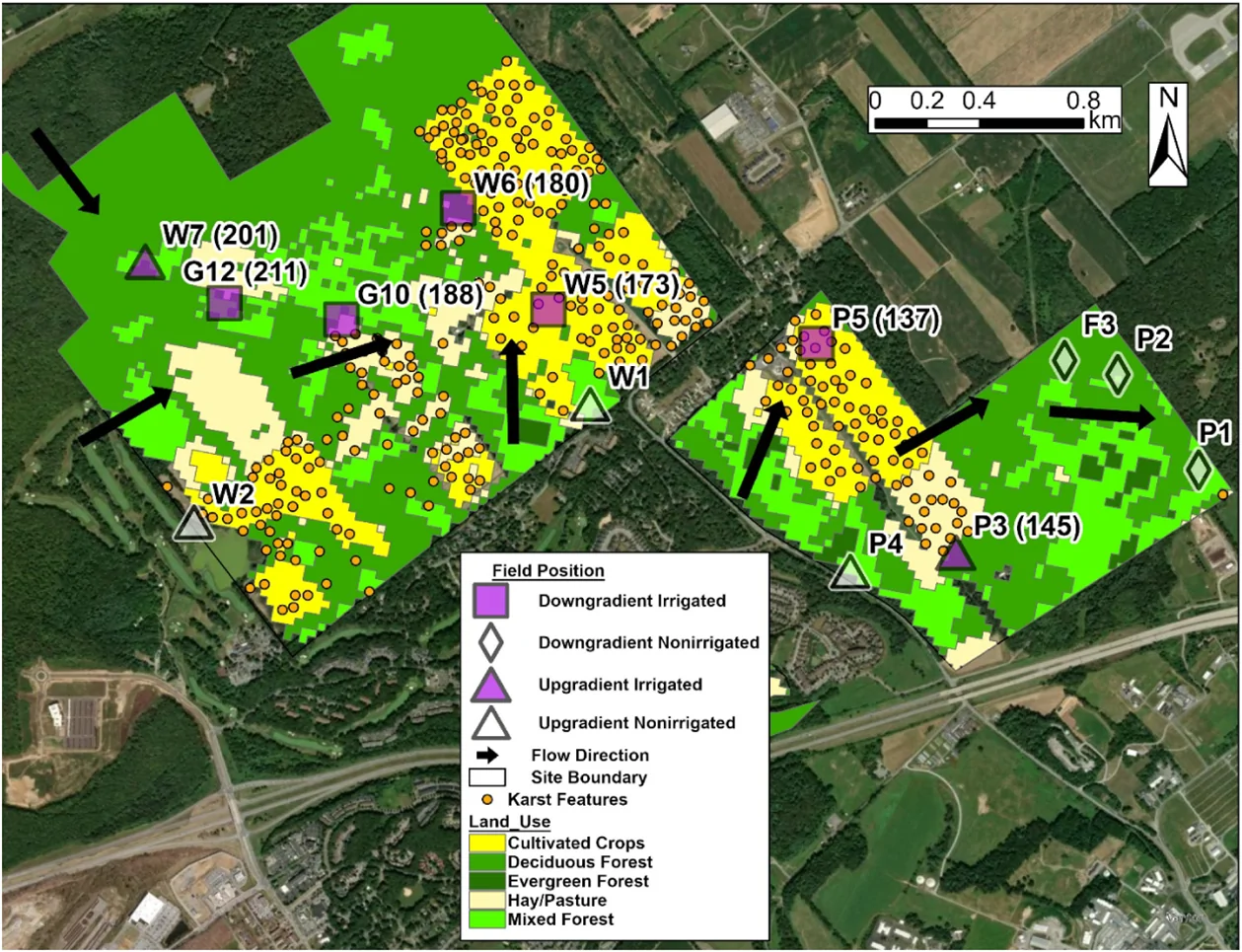
Study site map showing well field positions, average annual irrigation depths (cm) given in parentheses next to well ID, surface karst features,^21^ and cropped versus forested land use areas.

The Pennsylvania Department of Environmental Protection (PA DEP) permits site irrigation of up to 5 cm ha^–1^ wk^–1^ of effluent, provided that groundwater nitrate levels remain below 10 mg-N/L to protect nearby drinking water supply wells. To this end, 13 groundwater monitoring wells, located upgradient and downgradient relative to the irrigation laterals (shown in Supplemental Figure S1), are used to capture spatial variations in groundwater elevation and quality (Figure 1).

### 2.2 Effluent and Groundwater Sampling

The wastewater effluent has been sampled biweekly for ammonia, nitrate, nitrite, and total Kjeldahl nitrogen (TKN) by staff at the Water Reclamation Facility (WRF), with effluent data subsetted to match the months of groundwater sampling. Effluent sampling for PFAS was done simultaneously with groundwater sampling, with bimonthly samples collected between 2019 and 2023 for a total of 18 sampling events. Samples were analyzed for 20 PFAS using EPA Method 537.1 ^17,22^ and nitrate using EPA method 300.0/2.1.^23^ Pharmaceutical sampling was conducted from 2016 to 2017 for a total of 11 sampling events of groundwater and wastewater effluent that were extracted for seven pharmaceuticals (acetaminophen, ampicillin, caffeine, naproxen, ofloxacin, sulfamethoxazole, and trimethoprim).^19^ Field blanks were taken and average detectable values were used to censor concentrations. Detection limits for all pharmaceuticals was 0.1 μg/L except ofloxacin, which was 0.3 μg/L.^19^ Additional methodology descriptions are given in the Supporting Information (Supplemental Text S2). Only the nine PFAS with quantifiable concentrations were included in this current study: perfluorobutanoic acid (PFBA), perfluorobutanesulfonic acid (PFBS), perfluoropentanoic acid (PFPeA), perfluorohexanoic acid (PFHxA), perfluorohexanesulfonic acid (PFHxS), perfluoroheptanoic acid (PFHpA), perfluorooctanoic acid (PFOA), perfluorononanoic acid (PFNA), and perfluorooctanesulfonic acid (PFOS). Detection limits for all PFAS ranged from 0.42 to 0.50 ng/L except for PFBA which ranged from 1.80 to 1.90 ng/L.

### 2.3 Hydrologic and Geospatial Data

Site-specific factors such as rainfall, land use, karst features, groundwater elevation, irrigation amounts, recharge estimates, soil property data, wastewater loads, and concentration data were utilized to explain differences in groundwater contaminant variability and transport through correlation analysis and the DRASTIC-L model (Section 2.4.4). Sources of each data set and model parameters are given in Supporting Information Supplemental Text S3. Nonirrigated wells were defined as those at least 31 m (100 ft) beyond any spray-irrigation radius to satisfy the minimum protection radius for a drinking water well.^24^

### 2.4 Spatial and Statistical Analyses

#### 2.4.1 Software

ArcGIS Pro Version 3.3.1 was used to conduct the DRASTIC model and all other spatial analyses including land use classification, karst feature proximity, irrigation assessment, and in the development of the site maps that visualize the results of this study. All statistical analyses used Rstudio Version 4.4.3 with the packages “tidyverse” and “dplyr” for data manipulation and analysis,^25,26^ “lubridate” for time series analysis,^27^ and “ggplot2” for visualization.^28^

#### 2.4.2 Spatial Variability

The occurrences of PFAS, pharmaceuticals, and/or nitrate were determined by the detection frequency (i.e., % of samples with detectable concentrations) for each well for each compound. Detection frequency correlations were evaluated using Spearman’s rank correlation coefficient (ρ-rho) and well-specific concentration boxplots were then used to identify spatial variabilities and infer similarities in environmental fate and transport dynamics among PFAS, pharmaceuticals, and nitrate. All nondetects (ND) were treated as zero for the calculations of detection frequency to avoid inflation of detection frequencies.^29^ Furthermore, spatial variability was also evaluated using a cumulative contaminant ranking by summing all concentrations above the detection limit for each well then ordering them from highest cumulative impact to lowest.

#### 2.4.3 Risk Calculations

While previous risk assessments at this site focused on livestock feed rations and food chain implications,^17^ the current study evaluates PFAS concentrations that exceed the EPA's proposed drinking water standards for the six compounds included in the 2024 primary drinking water regulation: hexafluoropropylene oxide dimer acid (HFPO–DA), PFBS, PFOA, PFOS, PFNA, and PFHxS. These exceedances were used to assess potential management and regulatory implications.^30^ However, all regulations are likely to be rescinded except for PFOA and PFOS.^31^ Pharmaceuticals do not have an equivalent regulatory criterion. Previous research at this site used Drinking Water Equivalent Levels to calculate human health risk quotients but found little risk to human health.^19^ Therefore, Predicted No Effect Concentrations for antimicrobial Resistance (PNEC-R) values^32^ (Supplemental Table S1) were chosen to address a gap in previous assessments by accounting for the potential risk of antimicrobial resistance.^33^

#### 2.4.4 Hydrogeologic Transport Drivers

Given the site’s variable hydrogeologic and land-use conditions, groundwater vulnerability of each well was mapped using the DRASTIC method modified for land use (DRASITC-L).^34,35^ The DRASTIC-L method is a standardized approach for evaluating relative, regional groundwater vulnerability considering seven hydrogeologic parameters: (D) depth to water table; (R) recharge (precipitation and irrigation), (A) aquifer media, (S) soil media, (T) topography, (I) impact of the vadose zone, (C) hydraulic conductivity, and (L) land use. Each of these parameters are assigned a weight and rating then summed to equal the Drastic Index (DI).^35,36^ A higher DI indicates an increased relative groundwater vulnerability. Typically, nitrate data are used to evaluate DRASTIC model performance, and only one study has evaluated PFAS performance using DRASTIC.^37^ The modified land use method was used because of recent reports indicating higher concentrations of PFAS in water supplies surrounding agriculture and developed land.^38–40^ This method is suitable for sites with an area of at least 40.5 ha.^34^ Rating and weight criteria were adapted from Tsegay et al.,^35^ and map color classes were adapted from Patel et al.^34^ and can be viewed in Supplemental Table S2. Indices were categorized into equal intervals: very low, low, moderate, and high.^41^ The model was validated at each well by correlating DI with cumulative contaminant ranking of each contaminant type. Futhermore, to account for DRASTIC-L’s inability to model groundwater flow, we utilized a multiple linear regression model that predicts cumulative contaminant ranking using a combination of the DI, hydraulic gradient position (upgradient or downgradient) and irrigation intensity.

## 3 Results and Discussion

### 3.1 Detection and Distribution

Over the 18 sampling events from 2019 to 2023, the total sum of detectable PFAS and nitrate exhibited high maximum detection frequencies across the site. The total sum of detectable PFAS detection frequencies reached 90% in wells G10, W6, and P2 and 100% for nitrate in wells G10, W6, P3, P5, W5, P2, and P1. Well-specific maximum detection frequencies of the nine individual PFAS ranged from 0 to 100%, with PFBS, PFHxA, and PFOA showing 100% detection across all wells. In contrast, across the 11 sampling events in 2016–2017, pharmaceuticals showed consistently lower detection frequencies, with a maximum detection frequency of 34% in well P2. Sulfamethoxazole had the highest maximum detection frequency (82% in wells F3, P1, P2, and P5) and ampicillin had the least (maximum detection frequency of 20% in well W2). Furthermore, no significant differences in occurrence were observed between PFAS chain length (short versus long) or functional group (carboxylic versus sulfonate acid), which likely reflects the site’s long-term application history (60+ years) (Supplemental Figures S2–S4). Similar research on sites with historical contamination have found groundwater concentrations comprised proportionally of long and short chain compounds (Sadia et al., 2023).^42^

Spearman’s correlation coefficients revealed positive associations among the detection levels for nitrate and all nine PFAS, with rho values ranging from 0.40 to 0.86. PFOA, PFHxA, and PFBS correlated strongly (ρ = 1) with each other, indicating consistent simultaneous detection and suggesting shared transport behavior (Supplemental Figure S5). Similarly, PFHpA and PFHxS were correlated strongly (ρ = 1). Pharmaceuticals often showed weak or no correlations with nitrate and PFAS, with the exception of sulfamethoxazole, the most frequently detected pharmaceutical. Pharmaceuticals also showed weak or no correlations with each other except for a few instances.

The more consistent detections of PFAS and nitrate than pharmaceuticals align with previous studies that found nitrate and PFAS can persist in groundwater for many years.^42,43^ Pharmaceuticals can be more prone to degradation,^44^ with reported half-lives for the compounds in this study spanning from 35 hours up to 4 years.^19^ No significant correlations were found between pharmaceutical half-life and detection frequency. This may suggest that persistence alone does not control pharmaceutical occurrence; instead, detection patterns may be more strongly influenced by source loading (Section 3.3).

Contaminant concentrations across wells show differences in variability among contaminant types (Figure 2). Nitrate concentrations exhibited less variability (coefficient of variation (CV) of 0.51) compared to PFAS and pharmaceuticals (CV of 0.66 and 2.83, respectively). PFAS concentrations showed broader concentration ranges that spanned over 2.43 orders of magnitude (0.58–156.7 ng/L), with widespread occurrence across wells (Figure 2). Pharmaceuticals displayed the greatest variability that spanned 3.36 orders of magnitude (0.05–114.94 μg/L), with low median values in most wells but high concentrations in several (Figure 2). These outliers were mainly driven by naproxen and ofloxacin, which had concentrations as high as 98.39 and 114.94 μg/L, respectively. The low variability of nitrate may represent nitrate’s highly soluble, poorly sorbed behavior whereas PFAS and pharmaceuticals exhibit diverse sorption characteristics and pharmaceuticals exhibit complex degradation kinetics.

**Figure 2:**
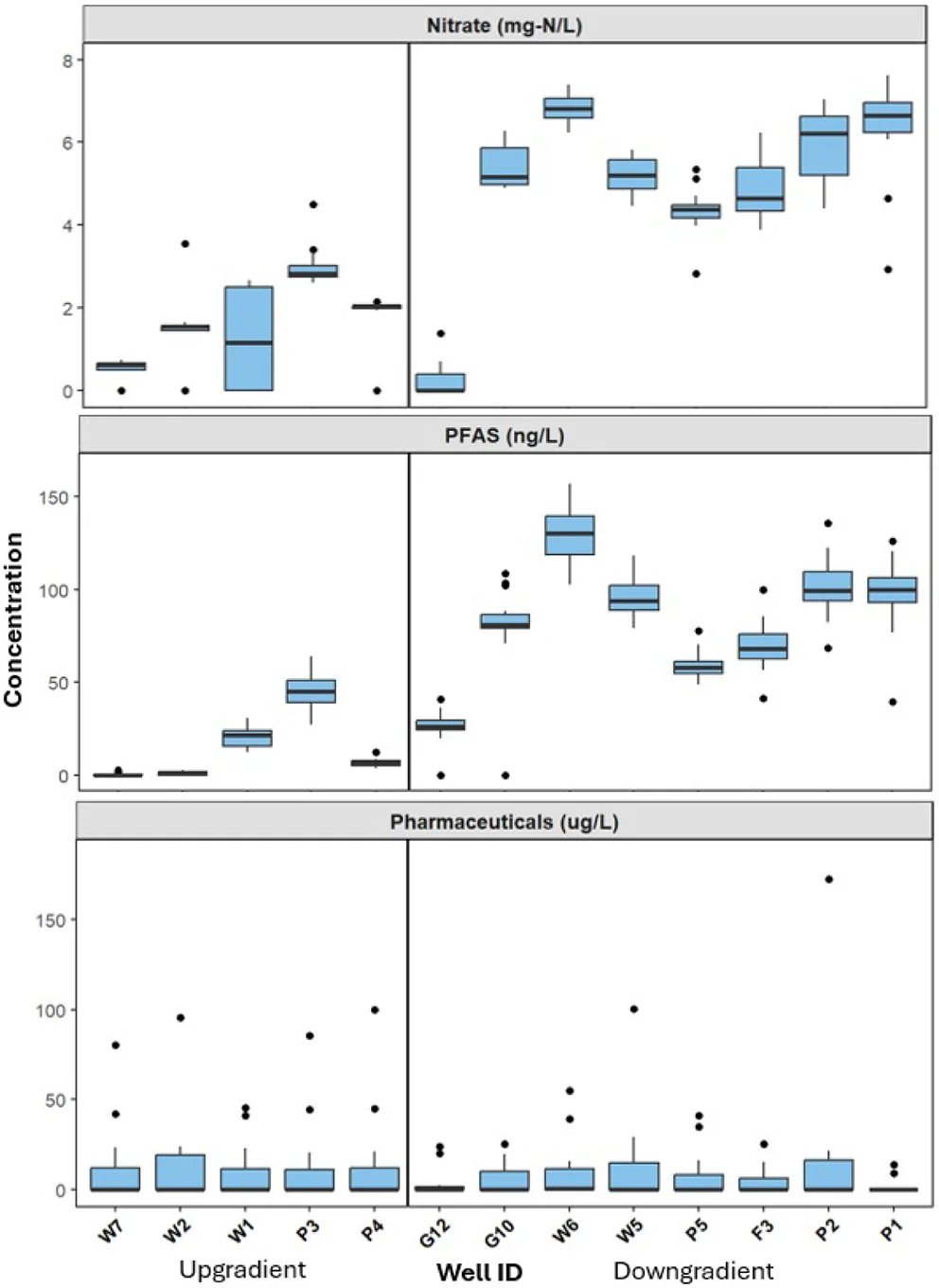
Boxplots of contaminant concentrations for each monitoring well at the spray-irrigation site, with wells categorized as upgradient or downgradient of irrigation activities by the solid vertical line in each graph. Boxes represent the interquartile range (25th–75th percentile), horizontal lines indicate the median, whiskers extend to 1.5× the interquartile range, and points represent outliers.

Wells upgradient of irrigation (P3, W7, W1, W2, and P4) generally showed lower concentration (ranges and median values) for PFAS and nitrate, while wells downgradient of irrigation activity generally showed higher concentrations (Figure 2). However, this pattern was not observed for pharmaceuticals (Figure 2), which showed similar ranges and median values for all wells, indicating a weaker spatial gradient likely due to pharmaceutical sorption, biodegradation, and dilution in the vadose zone.^19^

### 3.2 Well-Specific Variability and DRASTIC Index (DI)

Cumulative contaminant ranks in each of the wells are shown alongside DI in Figure 3. Rankings were within one or two ranks of each other for PFAS and nitrate, with a correlation coefficient of 0.92. In contrast, cumulative contaminant rankings for pharmaceuticals often did not correspond to the rankings for nitrate and PFAS (ρ = −0.121 for PFAS and −0.0165 for nitrate).

**Figure 3:**
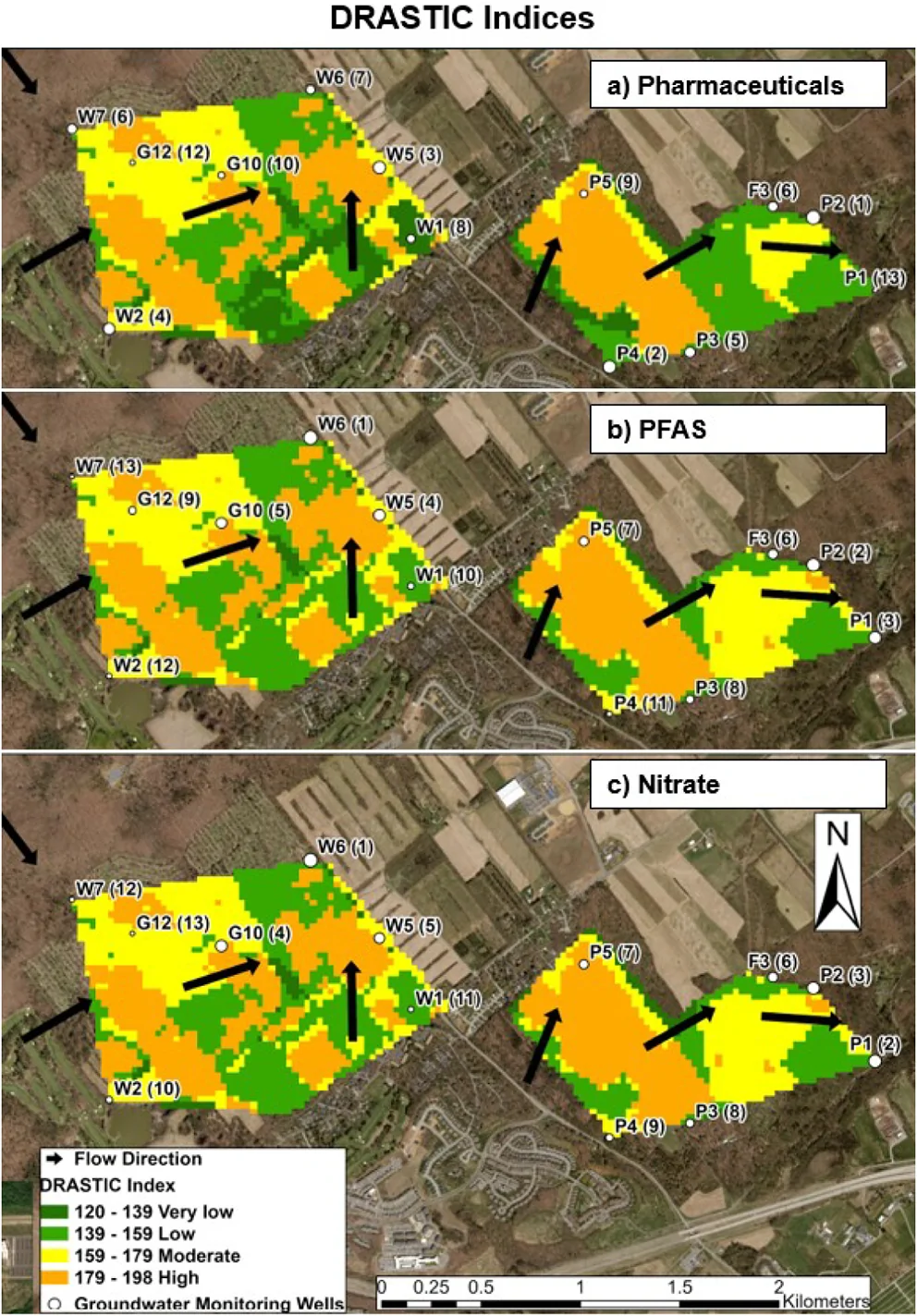
Mapped cumulative contaminant ranks based on the total sum of the sampling events (given in parentheses next to well ID) where 1= most impactful and 13 = least impactful, and DRASTIC indices by color: (a) Pharmaceutical sampling period (2016–2017); (b) PFAS sampling period (2019–2023); (c) Nitrate sampling period (2019–2023). Groundwater flow direction is given by the black arrows.

Areas with a higher DI often had higher cumulative contaminant rankings for PFAS and nitrate (Figure 3). DI generally exhibited positive correlation coefficients (ρ-rho) with PFAS and nitrate concentrations, and weakly negative coefficients with pharmaceutical concentrations. Nitrate had a ρ of 0.40 while PFAS ranged from 0.32 to 0.59 depending on the compound (Supplemental Table S3). Wells that ranked within the top five for cumulative PFAS and nitrate contamination also scored within the top five highest DI, with the exception of Well W6. Although Well W6 had a comparatively lower DI (144), it had the highest cumulative contaminant ranking (1) for PFAS and nitrate, potentially reflecting DRASTIC’s limitation of not accounting for groundwater flow. Well W6 is downgradient of heavily spray-irrigation areas (average annual irrigation of 210 cm), likely increasing its susceptibility to contaminant occurrence despite its lower DI. DRASITC-L scores were inversely correlated with PFAS (ρ = −0.328) and nitrate (ρ = −0.402) rank numbers, meaning that higher scores correspond to elevated contaminant levels (rankings closer to 1).

Standalone DRASTIC-L scores account for a minor portion of the spatial variance (R^2^ = 0.092 for total PFAS; R^2^ = 0.130 for nitrate; R^2^ = 0.037 for pharmaceuticals) before accounting for groundwater flow and irrigation intensity (Supplemental Figure S7). However, DRASTIC-L used in conjunction with localized groundwater flow data and source inputs improves the R^2^ values considerably for total PFAS and nitrate (Figure 4a and b). The R^2^ value increases to 0.74 for total PFAS and 0.484 for nitrate. Furthermore, the predicted and observed cumulative contaminant rankings show strong agreement (Figure 4a and b). The correlation coefficient (r) improved from 0.30 to 0.86 for total PFAS, and from 0.36 to 0.70 for nitrate after accounting for groundwater flow and irrigation intensity (Figure 4a and b; Supplemental Figure S8). Meanwhile, the model for pharmaceuticals does not follow a similar pattern, exhibiting an R^2^ of only 0.176 and moderate agreement between observed and predicted values (r = 0.42) (Figure 4c).

**Figure 4:**
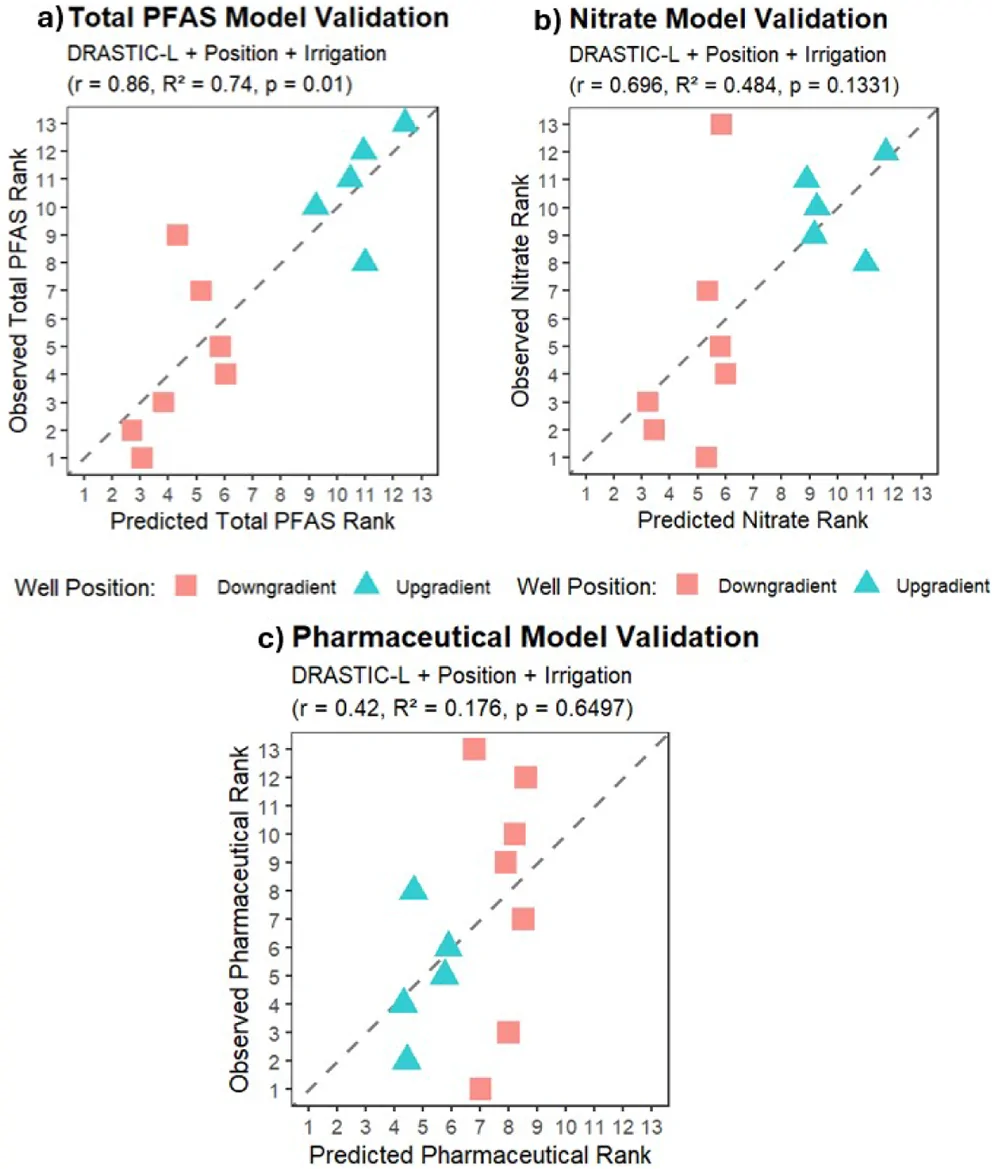
Observed versus actual cumulative contaminant rankings for (a) Total PFAS; (b) Nitrate; and (c) Pharmaceuticals. Correlation strength (r), goodness-of-fit (R^2^), and *p*-values are given.

The observed similarities between PFAS and nitrate responses to the DRASTIC-L model used in conjunction with groundwater flow information suggest this model’s applicability as a first approximation of relative vulnerability for certain anionic PFAS and may provide insights for monitoring priority selection. DRASTIC model results in previous studies have guided land use allocations for development and agriculture as well as located best management practices (BMPs) implementations to prevent groundwater pollution.^45,46^ Additionally, since biodegradability/biotransformationis low for both PFAS and nitrate, the DRASTIC model may be more suitable for more persistent compounds. This is due to DRASTIC-L’s limitations of not being able to account for biodegradation and biotransformation processes. Additional validation is needed for DRASTIC-L models due to limited sampling sizes.

### 3.3 Source Loading from Wastewater Effluent

Pharmaceutical occurrence in the wastewater effluent was variable and sporadic, likely indicating seasonal consumer usage.^19,47,48^ Naproxen (log K_ow_ = 3.18), ampicillin (log K_ow_ = 1.35), sulfamethoxazole (log K_ow_ = 0.89), and caffeine (log K_ow_ = −0.07) groundwater concentrations were often representative of recent wastewater inputs (Figure 5a and b).

**Figure 5:**
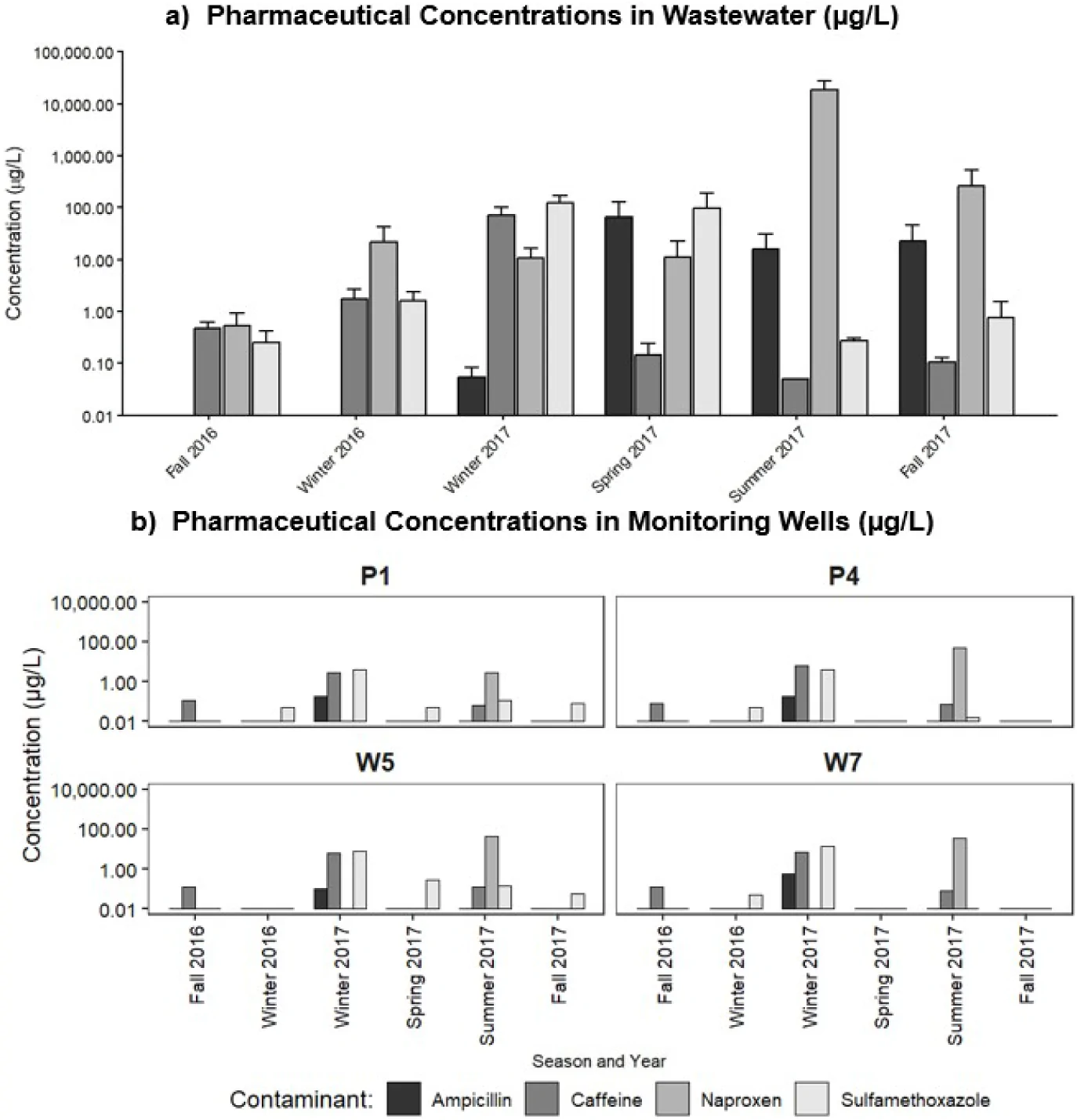
(a) Time series for wastewater effluent concentrations of pharmaceuticals (μg/L) and (b) Time series for well concentrations of pharmaceuticals (μg/L) in 4 representative wells (P1, P4, W5, W7).

Naproxen, a nonsteroidal anti-inflammatory (NSAID) widely used for treatment of inflammation, had the most prominent spike occurring in summer 2017. Given that the groundwater monitoring wells are 10–30 m deep, an immediate groundwater response is not expected. However, naproxen concentrations increased in the monitoring wells during the months when wastewater effluent concentrations were highest (Figure 5a and b). Moreover, after episodic increases in the groundwater, concentrations quickly returned to below the detection limit, suggesting that cumulative irrigation may not result in persistent groundwater concentrations at this site for compounds prone to degradation like naproxen. This short-term response of the groundwater may be due to the occurrence of preferential flow paths, which can increase the apparent hydraulic conductivity to several hundred meters per hour.^49,50^ Preferential flow at the study site has been well-documented^50^ and occurs at an increased frequency due to the high levels of irrigation activity. The increase occurrence of preferential flow due to irrigation is observed through the spikes in pharmaceutical concentrations that occur even in upgradient, unirrigated wells (such as P4 and W7) (Figure 4) which is reflective of the site’s high transmissivity and groundwater mounding.^51^

Furthermore, many of these wells have a surface karst feature (i.e., depression, sinkhole, etc.) within a 100 m radius, and some wells such as P5 and W5 have as many as 8 and 5 karst features within a 100 m radius (Figure 1).

Ofloxacin did not behave similarly to the other pharmaceuticals, likely due to its unique chemical structure. Ofloxacin is a common antibiotic classified as a fluoroquinolone and has a fluorine atom attached to its aromatic ring. The presence of this carbon–fluorine bond is known to contribute to its chemical stability and environmental persistence^52,53^ with a relatively long, four-year half-life in soil.^19^ Similar to long-chain PFAS, which also have decreased mobility in soils, ofloxacin presence in the wells may represent long-term storage dynamics more so than source inputs (Section 3.4).^54^

For PFAS, only PFPeA had strong, consistent significant positive correlations between groundwater and wastewater loads for 11 wells (correlation coefficients ranged from 0.49 to 0.71). All other PFAS showed either negative correlations or no significant correlation, indicating they did not follow similar trends to wastewater loads. In addition to wastewater, pesticide applications can also contribute to PFAS concentrations in groundwater; however, the groundwater underlaying cultivated cropland that receives pesticides did not exhibit higher PFAS than other land use types at the site (Supplemental Figure S6).

Similar to PFAS, nitrate concentrations in the monitoring wells had no significant correlations with wastewater loads of total nitrogen. Average seasonal total nitrogen loads applied by the effluent irrigation were highest in the fall (2654 kg) and lowest for the summer (818 kg). When converted to kg/ha, this is equivalent to 10.8 kg/ha for fall and 3.34 kg/ha for summer. About half of this site is cropland and receives fertilizer twice a year. Synthetic fertilizer application rates of nitrogen are about 5–12× higher than wastewater application rates of nitrogen. Therefore, nitrogen inputs at the site are likely heavily influenced by fertilizer application rates.

Monitoring wells had relatively stable nitrate concentrations across the seasons (Supplemental Table S4) and did not display any source-specific patterns in fertilizer application months. Furthermore, wells located in crop and pastureland use areas did not have elevated concentrations. Results suggest, similarly to PFAS, nitrate levels in the wells do not broadly reflect input rates and timing. This is in line with previous research that found nitrogen levels in groundwater reflect cumulative input rather than recent applications.^55^ To contextualize this cumulative signature, wells located upgradient of the spray fields within forested catchments (W1 and P4) were used as a proxy for background conditions. These wells exhibited lower median concentrations (2.04 and 2.49 mg-N/L for P4 and W1, respectively) (Figure 2) and were close to the 1.13 mg-N/L background level predicted for this region.^56^

### 3.4 Hydrogeologic Influences on Groundwater Concentrations

#### 3.4.1 Effect of Groundwater Elevation Dynamics on Contaminant Transport

Wells with significant, positive correlations with groundwater elevation were often downgradient of irrigation (Figures 1, 5, and 6). Three wells (W1, P4, and W2) showed significant negative relationships between PFAS concentration and groundwater elevation. These wells are positioned upgradient, and the inverse relationship with recharge may reflect the low background levels of PFAS concentrations in these wells. They have relatively low concentrations compared to other wells (Figure 2) and rank very low in cumulative exposure (Figure 3). One downgradient well, P1, also displayed significant, negative correlations with three compounds (PFOA, PFHpA, and PFHxS) (Supplemental Figure S11). Although P1 is a downgradient well, the significant negative correlations may be explained by its proximity to a nearby intermittent stream where stream infiltration may dilute PFAS concentrations.^57^

**Figure 6:**
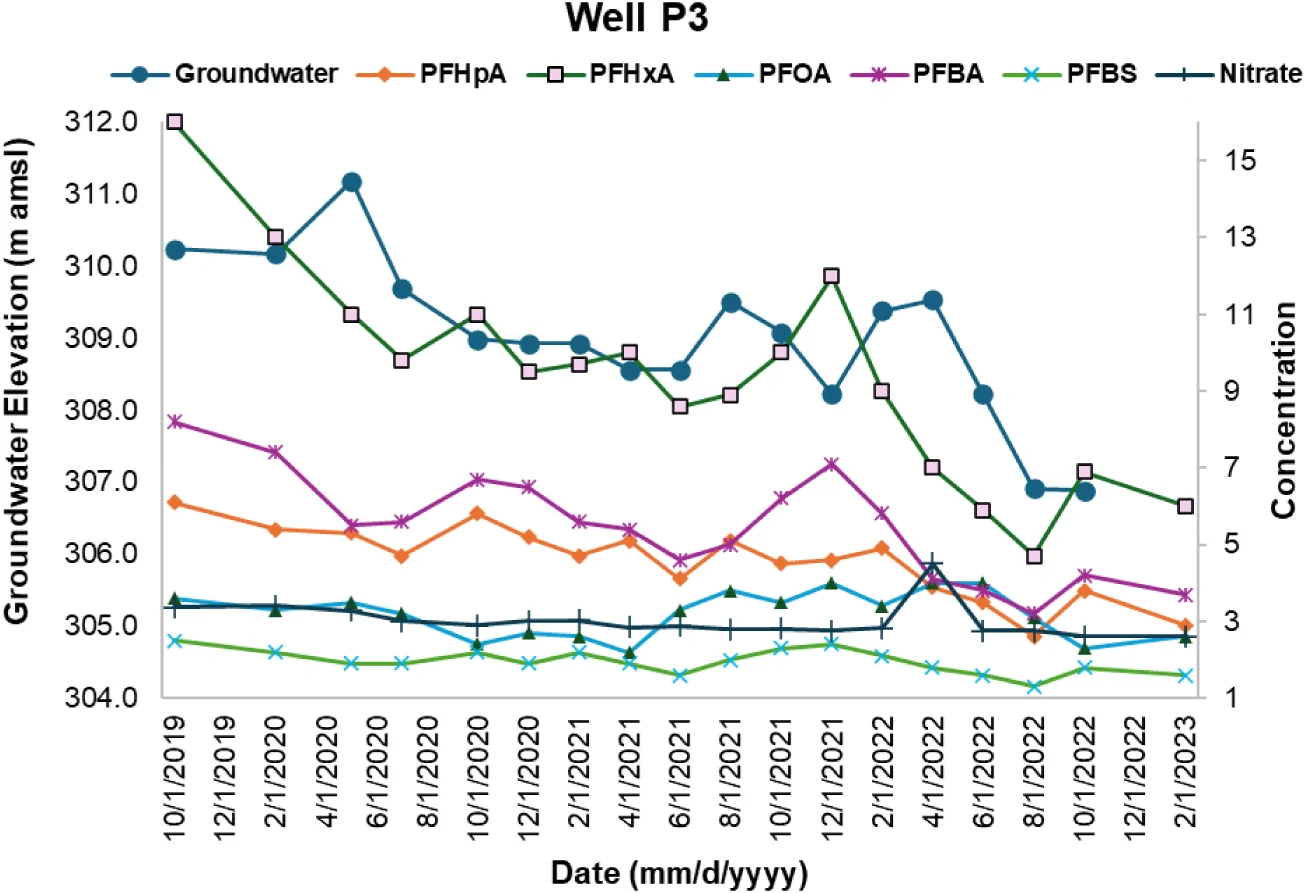
Time series of groundwater elevations in meters above sea level (m asml), nitrate concentrations (mg-N/L) and PFAS concentrations (ng/L) in well P3.

PFHxA, PFHpA, PFOA, PFHxS, PFOS, PFBA, PFNA, and PFBS all showed significant, positive correlations between concentrations and groundwater elevations in many of the wells. Positive correlation coefficients ranged from 0.54 to 0.86 depending on the compound. PFHxS exhibited the most consistent positive associations, showing significant correlations in four of the wells (G12, W6, P5, and W5). PFHxA was the second highest showing significant correlations in three of the wells (G12, P3, and P5). This is opposite of the finding from Mroczko et al.^17^ which found negative correlations with total PFAS and groundwater elevation. However, that specific analysis was limited to total PFAS over a shorter time frame, whereas we evaluated individual compounds using an extended data set.

Only PFPeA displayed consistent negative correlations among the PFAS class despite well position. PFPeA concentrations in six monitoring wells were significant and inversely related to groundwater elevation. PFPeA is a short-chain PFAS, and therefore appears to be less influenced by the factors known to govern long-chain PFAS, such as partitioning at the air–water interface.^58^

Nitrate exhibited similar trends to PFAS with significant negative correlations in wells P4 (−0.50) and W1 (−0.68) and significant positive correlations in wells G10 (0.80), W6 (0.81), and P3 (0.63) (Figure 6; Supplemental Figures S9–S14). The co-occurrence of similar correlation patterns for nitrate and PFAS in the same wells suggests shared controls on their transport dynamics. In fact, karstic systems exhibit both positive and negative associations between well concentrations of PFAS and nitrate and water table levels depending on well-specific concentrations relative to recharge concentrations.^59^ Positive associations between nitrate and water levels have been attributed to fracture activation and recharge.^60–62^ Positive associations between water level and PFAS concentrations are consistently reported in literature.^12,63,64^ As recharge increases water level, colloid release and air–water interface desorption contribute to PFAS mobility in groundwater.^58,63^ PFAS and nitrate have shown long-term storage, with agricultural land use serving as a sustained source for both at the site.^62,65,66^

#### 3.4.2 Effect of Rainfall on Contaminant Transport

Nitrate and PFAS showed generally weak and negative correlations with recent rainfall. Although not statistically significant, pharmaceuticals as a class showed the strongest correlations between rainfall and monitoring well concentrations. The strongest correlations were with antecedent 14-day rainfall with coefficients ranging from 0.24 to 0.75 depending on the well. Caffeine, naproxen, sulfamethoxazole, and trimethoprim were most consistently positively correlated with rainfall. Research on the impact of rainfall on groundwater concentrations of pharmaceuticals is generally limited. Bexfield et al.^67^ reported higher detections in shallower wells because of recent recharge. At our site, rainfall may facilitate faster transport to groundwater through preferential flow paths, in which pharmaceuticals respond more to surface inputs (wastewater effluent and rainfall) than groundwater elevation. As a result, routine quarterly sampling may not capture short-term spikes in pharmaceutical concentrations that are associated with irrigation or rainfall-driven recharge. Future research should focus on targeted sampling during and after rainfall events and periods of high pharmaceutical usage to better characterize the timing and magnitude of these inputs to groundwater.

### 3.5 Risk Calculation Implications for Management

#### 3.5.1 Exceedances of PNEC-R for Antibiotic Resistance

Every monitoring well showed exceedances of PNEC-R (for antibiotic resistance) thresholds for at least one antibiotic (sulfamethoxazole, trimethoprim, ofloxacin, or ampicillin); however, most wells only had a single exceedance. Sulfamethoxazole had the most frequent exceedances of PNEC-R thresholds (15% of samples; *n* = 142). Exceedances of sulfamethoxazole strongly coincided with periods of elevated effluent inputs: both concentrations and loads peaked in February, which was also the only month in which groundwater exceedances were observed. Ampicillin exceedances similarly coincided with wastewater peaks, with both occurring in the early months of 2017.

Trimethoprim exceedances did not coincide with effluent peaks, which is consistent with earlier results showing no significant correlation between trimethoprim in effluent and groundwater (Section 3.3). In general, trimethoprim was present at low concentrations (typically <15 μg/L in the wastewater effluent) and only 3% of total samples (*n* = 142) exceeded the PNEC-R threshold. Moreover, trimethoprim has a relatively long half-life in soil compared to the other compounds (62 days),^19^ so groundwater concentrations may be more influenced by previous inputs when compared to other compounds. Ofloxacin only displayed exceedances in the month with the highest groundwater elevation, which aligns with our expectations given its fluorinated chemistry and stronger sorption to soils. Wells that had more than one sample above the PNEC-R thresholds were in more heavily irrigated areas (wells G10 and G12).

If pharmaceutical concentrations remain below drinking water human health thresholds,^19^ then management should emphasize minimizing the potential for antibiotic resistance selection in groundwater supplies. This management approach would have to focus on irrigation load management, avoiding high volume applications during periods of wastewater effluent peaks and after high intensity rainfall events. For fluoroquinolones (such as ofloxacin), however, a management approach like that for PFAS might be needed.

#### 3.5.2 Exceedances of PFAS Drinking Water MCLs

PFAS exceedances depended on the groundwater elevation and the specific well (Supplemental Table S5). Exceedances were observed for PFOA and PFOS in 9 wells and PFHxS in 4 wells. Exceedances decreased as elevation decreased, which followed the general trends observed between PFAS and groundwater elevation in Section 3.4.1 (Supplemental Figures S15–S24). This downward trend in concentration led to some wells meeting Federal MCLs with other wells likely to meet them if the downward trend continues.

At the site, nitrate has historically shown a similar pattern with a long-term decline despite the short-term fluctuations driven by groundwater table dynamics. Based on the most recent analysis, groundwater at the site meets the drinking water MCL for nitrate. Since PFAS and nitrate showed similar transport drivers, there is potential that the management strategies implemented at the site for nitrate are also effective for PFAS. To manage nitrate levels, the site implemented treatment plant upgrades and conservation efforts to lower flows into the treatment plant, and strategies that maintained infiltration rates (i.e., cover cropping, organic matter). Furthermore, total influent loading was reduced by separating community wastewater flows into two different treatment plants. These strategies coupled with the phase-out of long-chain PFAS likely helped contribute to the gradual decline in groundwater concentrations observed in the study, consistent with reported decreases in long-chain PFAS concentrations in wastewater effluent following voluntary phase-outs.^68^ Since there is no historic monitoring of PFAS, only nitrate, additional monitoring is recommended to validate whether the observed trends represent a long-term decline in groundwater concentrations. Results presented in this study could serve as a baseline for future studies. Additionally, it is unclear how most recent wastewater treatment facility upgrade impacts PFAS partitioning to the solid versus aqueous phase, which would change concentrations in the treated effluent.

## 4 Conclusions

Overall, PFAS management approaches with emphasis on groundwater elevation may be best suited based on the observed transport dynamics and patterns. The minimum elevations where exceedances begin should be considered (Supplemental Table S5) and increased monitoring to further evaluate the impacts groundwater elevation has on these exceedances. Additionally, the DRASTIC-L model used in conjunction with groundwater flow data may help identify higher vulnerability regions that can be targeted for more monitoring; although, more validation is needed.

In contrast, pharmaceuticals exhibited episodic occurrence driven by recent wastewater inputs and rainfall events with strong attenuation in the vadose zone and limited persistence in groundwater. As a permitted reuse site, irrigation activities are scheduled spatially and temporally to accommodate seasonal and intermittent rain events by favoring on forested land in nongrowing periods and accounting for hydrogeological variations within the site. This flexibility is critical in managing pharmaceuticals effectively.

## Supplementary Material



## Notes

Additional methodological details describing data set sources and the water reclamation facility (WRF), along with supplemental tables and figures presenting correlation analyses, seasonal nitrate concentrations, and PFAS exceedances in relation to groundwater elevation (PDF)
