# Supplementary material for: Hydrogeologic Drivers and Management Implications of PFAS, Nitrate, and Pharmaceuticals in Groundwater at a Karst Beneficial Reuse System
Source: ACS ES T Water. 2026 Jul 28;6(8):5377–86. doi: 10.1021/acsestwater.6c00547 (PMC13488393; doi:10.1021/acsestwater.6c00547)
Supplement: Supplementary file 1 [file ew6c00547_si_001.pdf]

Supplementary Materials

Hydrogeologic Drivers and Management Implications of PFAS, Nitrate, and Pharmaceuticals in Groundwater at a Karst Beneficial Reuse System

*Kelly Kosiarski[a], Tamie L. Veith[b], Faith A. Kibuye[c,d], Patrick J. Drohan[c], Onur G. Apul[e], Heather E. Preisendanz[a,f] **

[a] Department of Agricultural and Biological Engineering, The Pennsylvania State University, University Park, PA, 16802, USA

[b] USDA-Agricultural Research Service, Pasture Systems and Watershed Management Research Unit, University Park, PA, 16802, USA

[c] Department of Ecosystem Science and Management, The Pennsylvania State University, University Park, PA, 16802, USA

[d] Penn State Extension, The Pennsylvania State University, University Park, PA, 16802, USA

[e] Department of Civil and Environmental Engineering, The Pennsylvania State University, University Park, PA, 16802, USA

[f] Institute of Sustainable, Agricultural, Food and Environmental Science, College of Agricultural Sciences, The Pennsylvania State University, University Park, PA, 16802, USA

Number of Pages: 28
Number of Text Sections: 3
Number of Tables: 5
Number of Figures: 24

Supplemental Text S1. Description of the Water Reclamation Facility (WRF)

The water reclamation facility (WRF) is designed to treat influent flow from the Penn State University and a small percentage of the surrounding municipality (~3%). A brief history and description of the WRF is given by Kibuye et al. (2019)[1]. However, the WRF recently went through a major reconstruction, beginning near the end of 2018 and completed in 2024. The major upgrades included installation of a new headworks building, activated sludge tanks, a membrane bioreactor filtration system, and a sludge thickener, plus rehabilitation of the effluent pump stations. The permitted annual average design flow of the upgraded facility is 11.36 million liters per day (MLD) with an average annual flow of 6.06 MLD.

Supplemental Text S2. Methodology description for PFAS and Pharmaceutical Extraction

Pharmaceutical compounds were analyzed within 48 hours of sampling using a high performance liquid chromatography (HPLC) coupled with a Q Exactive mass spectrometer (from Thermo Fisher Scientific). An injection volume of 20 µg/L was used and eluted using an aqueous and methanol buffer. The mass spectrometer operated with alternating electrospray ionization (ESI) polarities in Full MS mode for quantification, while structural identification and confirmation were done in data-dependent MS^2 ($ddMS^2$) mode[1]. Per and polyfluoroalkyl substances (PFAS) were shipped overnight to Eurofins Lancaster Laboratories in Lancaster, PA and analyzed within 15-30 days of collection. Samples are analyzed following EPA method 537.1 which utilizes liquid chromatography and a triple-quadrupole mass spectrometer (LC-MS/MS). Solid phase extraction (SPE) was performed using polystyrene divinylbenzene (SDVB) cartridges to concentrate 250 mL of water. Samples were eluted with methanol, concentrated with nitrogen, and adjusted to a final volume of 1 mL in a methanol and water mixture containing internal standards. The mass spectrometer operated in negative ESI mode[2,3].

Supplemental Text S3. Sources for Datasets

Rainfall data were obtained from the Global Historical Climatology Network – Daily (GHCN-Daily), Version 3 dataset[4,5]. Dominant land use (either agriculture or forested) for each well location was determined using the National Land Cover Database (NLCD) from the Multi-Resolution Land Characteristics Consortium (2021)[6]. Proximity to nearest karst feature and number of karst features surrounding the wells were determined using a karst inventory dataset from the Pennsylvania Spatial Data Access[7]. Irrigation average annual totals (cm) for the laterals (Figure 1) were used in conjunction with rainfall to calculate recharge and relative impact of each well area[8]. Natural recharge for the area surrounding each well was estimated using data from the United States Geologic Survey[9]. Groundwater elevation at the time of sample collection for each well

was provided by Penn State University Office of Physical Plant staff. Soil data were obtained from the United States Department of Agriculture - Natural Resources Conservation Service (USDA-NRCS) Web Soil Survey[10]. To integrate groundwater monitoring well data with groundwater elevations and wastewater loads, the datasets were matched by month/year since the exact dates of sampling varied between datasets.

Supplemental Table S1. Predicted No Effect Concentrations for antimicrobial Resistance (PNEC-R) values for the antibiotic compounds in the study[11].

Compound	PNEC-R (μg/L)
Trimethoprim	0.5
Ampicillin	0.5
Ofloxacin	0.5
Sulfamethoxazole	0.6

Supplemental Table S2. DRASTIC Parameter weights and rating ranges for study location.

Parameter	Weight[a]	Site Specific Rating Ranges
Depth to Water 2016-2017	5	1-3
Depth to Water 2019-2023	5	1-4
Recharge	4	5-8
Aquifer Media	3	10
Soil Media	2	4-9
Topography	1	8-10
Impact of Vadose Zone	5	10
Hydraulic Conductivity	3	1
Land Use	5	2-8

[a] = Weights from Tsegay et al. (2024)[12].

Supplemental Table S3. DRASTIC Spearman's correlation coefficients.

Compound	Coefficient	P-Value
	**PFAS**	
PFBA	0.32	0.31
PFBS	-0.01	0.97
PFPeA	0.34	0.29
PFHxS	0.36	0.30
PFHpA	0.32	0.31
PFOA	0.34	0.28
PFOS	0.53	0.08
PFNA	0.59	0.04
	**Pharmaceuticals**	
Acetaminophen	-0.05	0.88
Ampicillin	0.08	0.80
Caffeine	0.06	0.86
Naproxen	-0.26	0.42
Ofloxacin	0.07	0.84
Sulfamethoxazole	0.03	0.93
Trimethoprim	-0.45	0.14
	**Nutrients**	
Nitrate	0.40	0.195

Supplemental Table S4. Seasonal nitrate concentrations (mg/L).

Season	Mean	Median	Standard Deviation	Number of samples
Fall	3.37	4.25	2.62	51
Spring	3.78	4.32	2.27	43
Winter	3.76	4.37	2.29	70
Summer	3.65	3.72	2.34	84

Supplemental Table S5. Well specific exceedances of PFAS drinking water standards (total samples; *n*=18) and minimum groundwater elevations at which exceedances were observed.

Well ID	Compound	Minimum Elevation (m)	% Exceedance
F3	PFOA	N/A	100
	PFOS	N/A	88.9
G10	PFHxS	311.2	50
	PFOA	311.2	94.4
	PFOS	311.2	94.4
G12	PFOA	311.0	83.3
P1	PFHxS	283.5	22.2
	PFOA	283.5	100
	PFOS	283.5	100
P2	PFHxS	303.3	27.8
	PFOA	295.8	100
	PFOS	295.8	100
P3	PFOA	306.9	100
	PFOS	309.1	5.6
P5	PFOA	307.0	100
	PFOS	307.0	94.4
W1	PFOS	314.3	61.1
W5	PFOA	310.1	100
	PFOS	310.1	100
W6	PFHxS	309.0	100
	PFOA	309.0	100
	PFOS	309.0	100

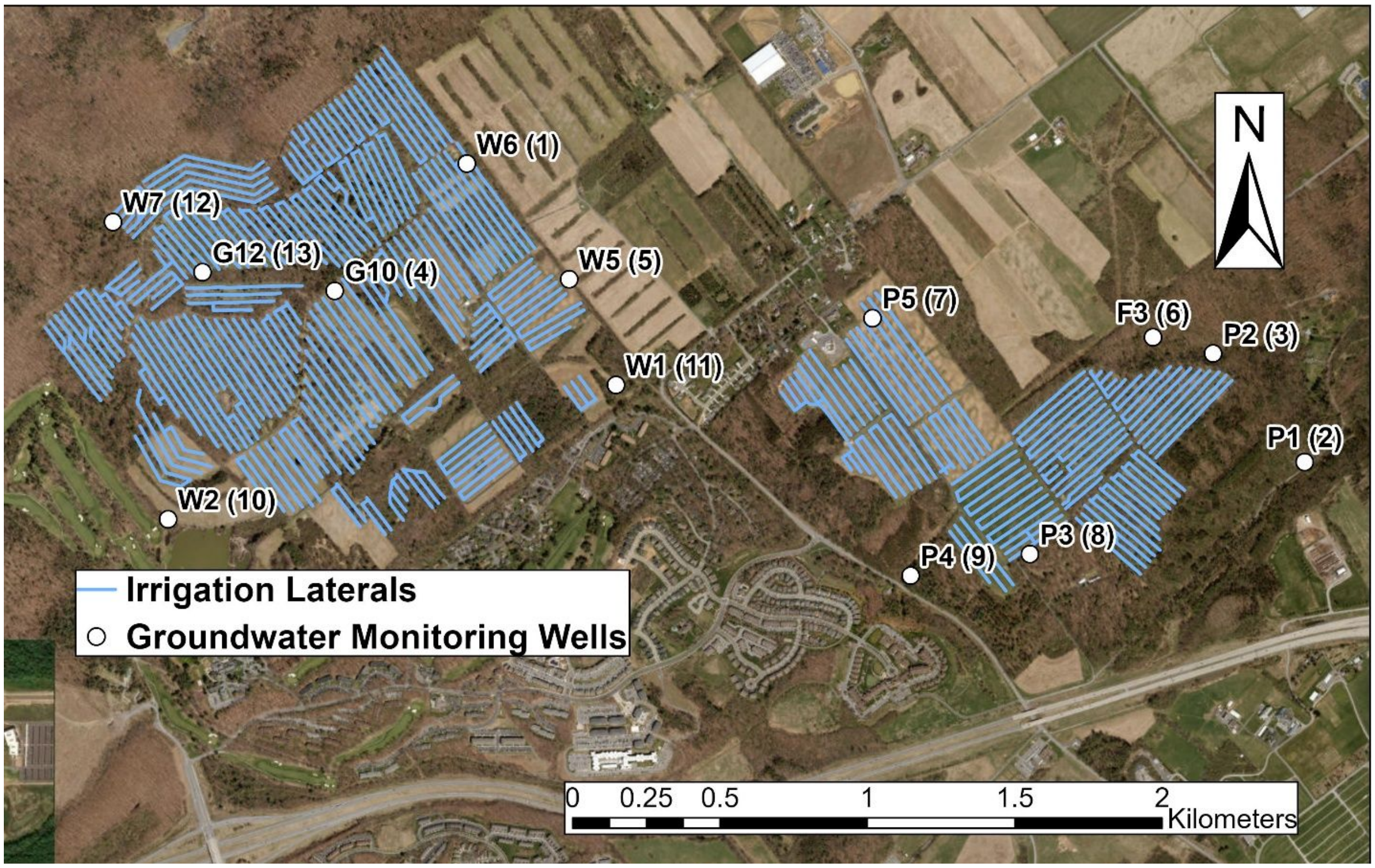

Supplemental Figure S1. Irrigation lateral map with Well IDs followed by average annual totals in cm for the laterals in parentheses.

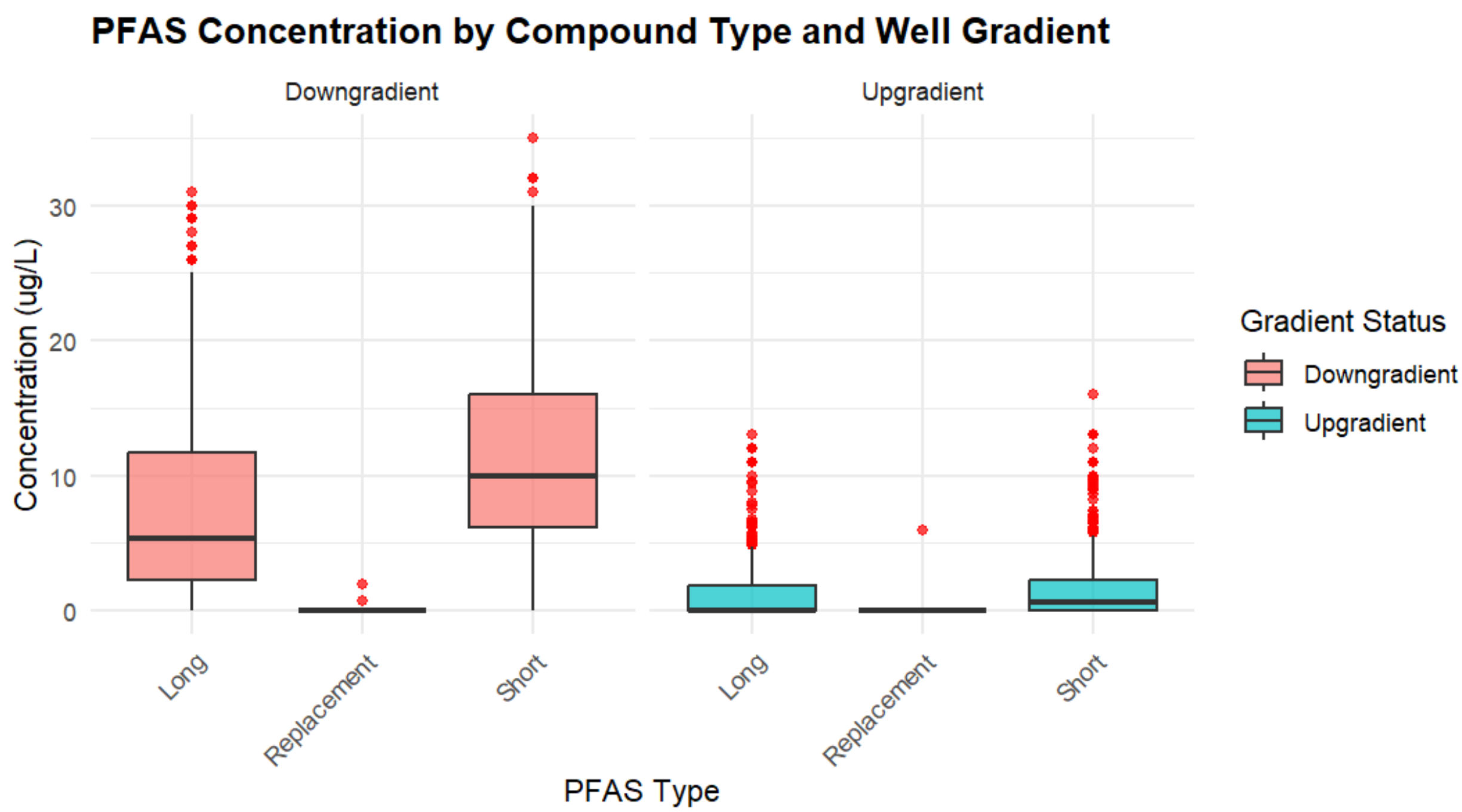

Supplemental Figure S2. PFAS concentration (ng/L) by chain length in upgradient and downgradient wells.

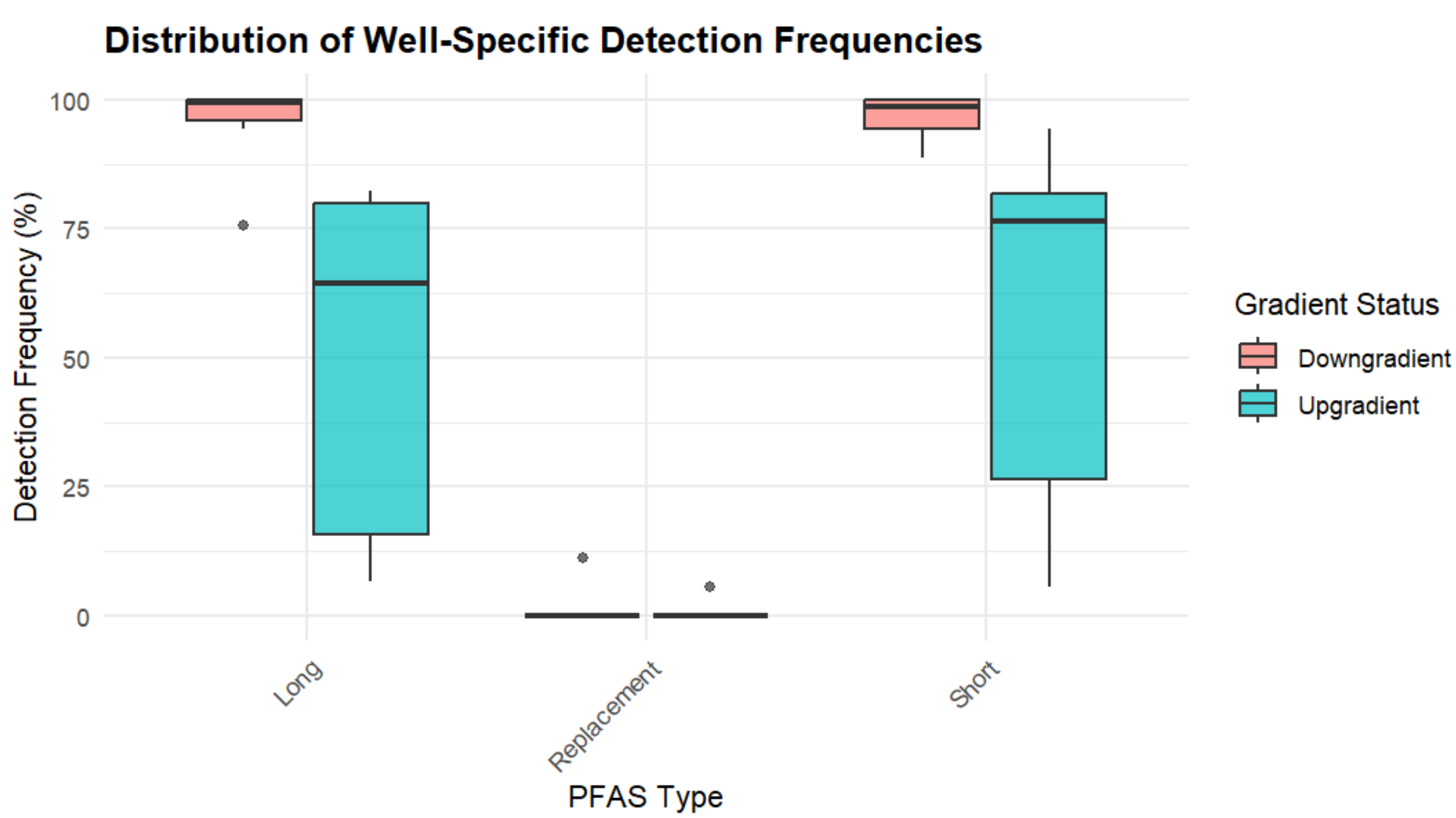

Supplemental Figure S3. PFAS detection frequency (%) boxplots by chain length in upgradient and downgradient wells.

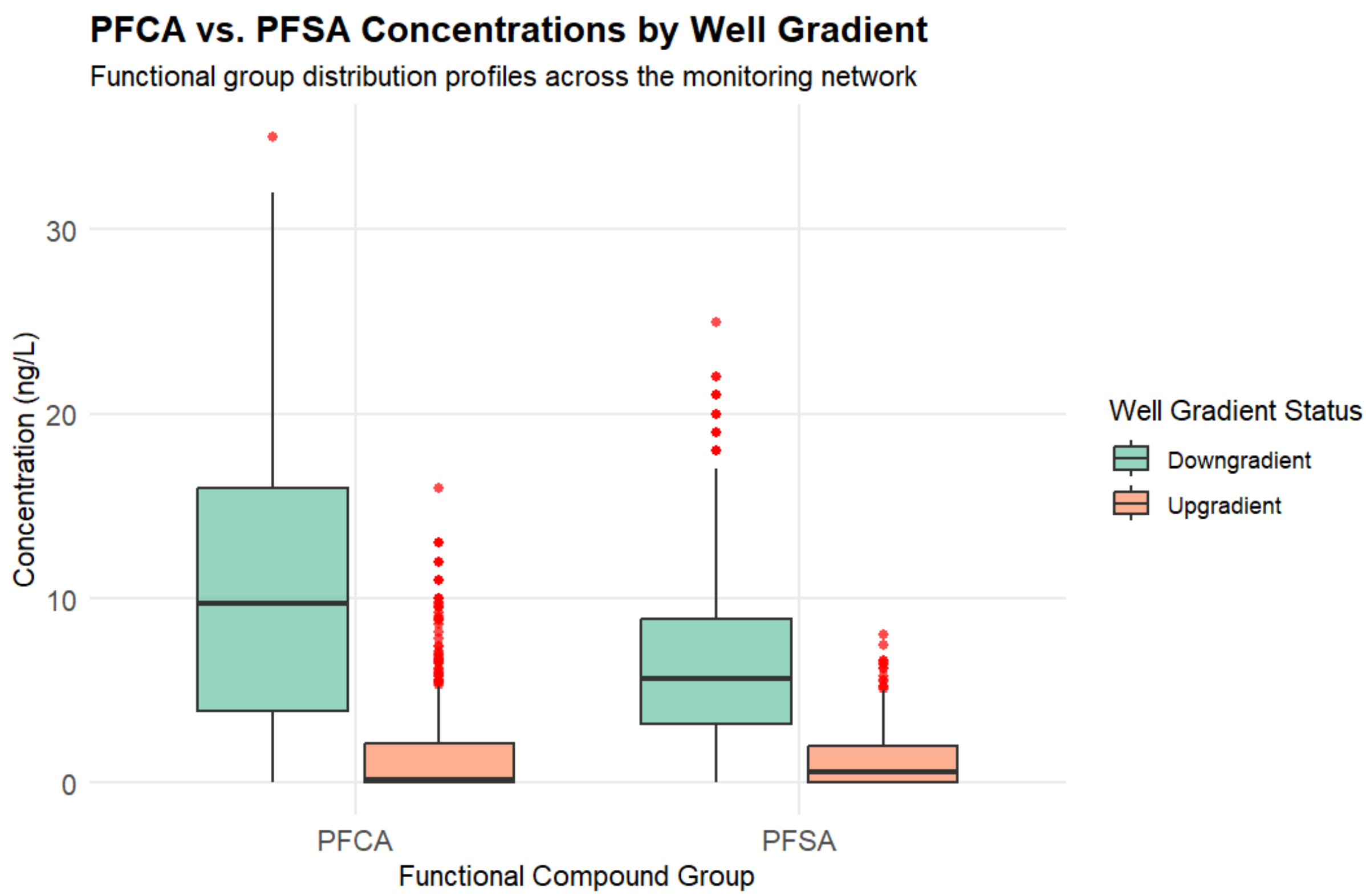

Supplemental Figure S4. PFAS concentration (ng/L) boxplots by functional group type in upgradient and downgradient wells.

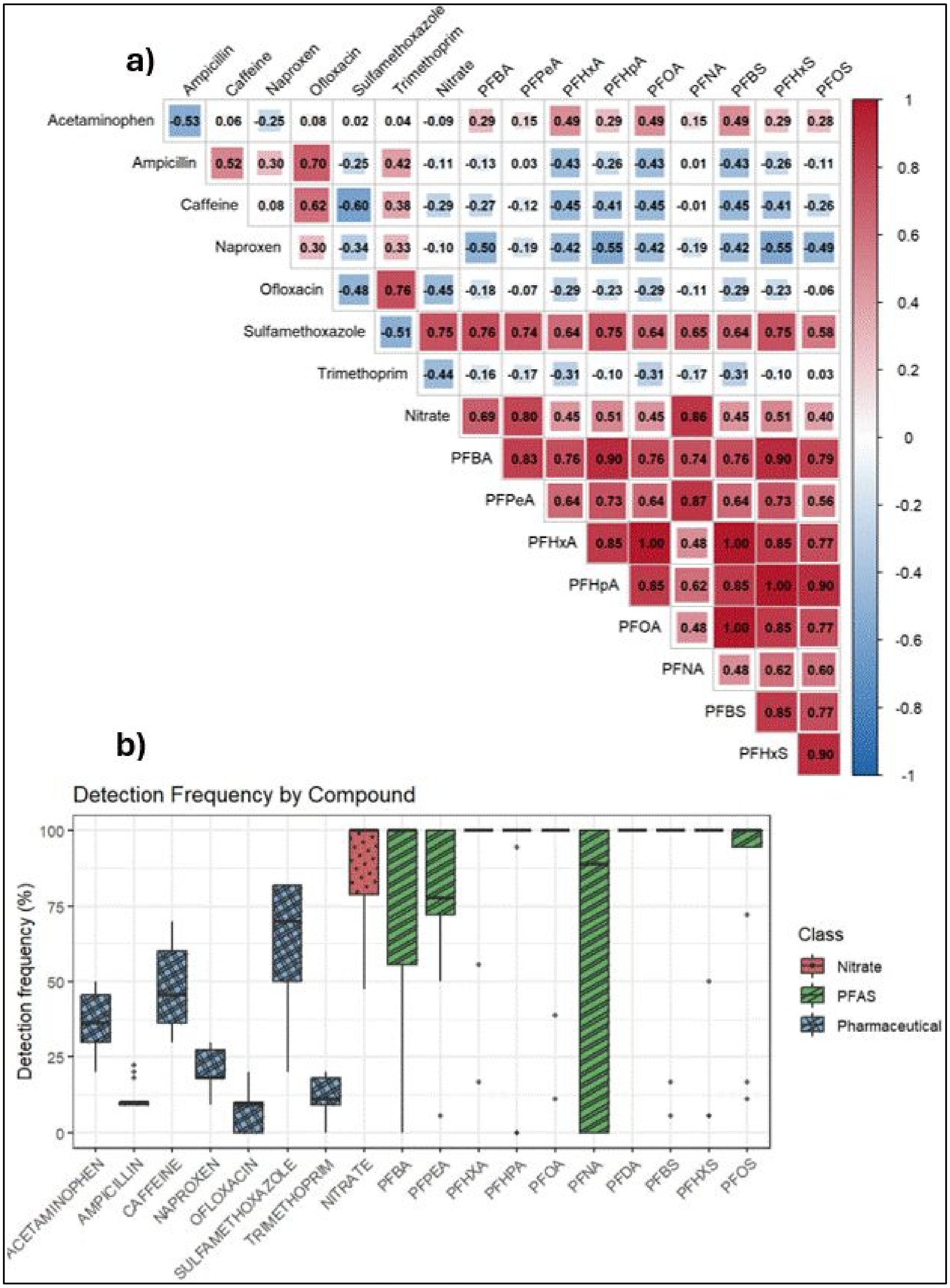

Supplemental Figure S5. Detection Frequency correlation plot and boxplots of detection frequencies in each of the 13 monitoring wells by compound (% of samples with detectable concentrations).

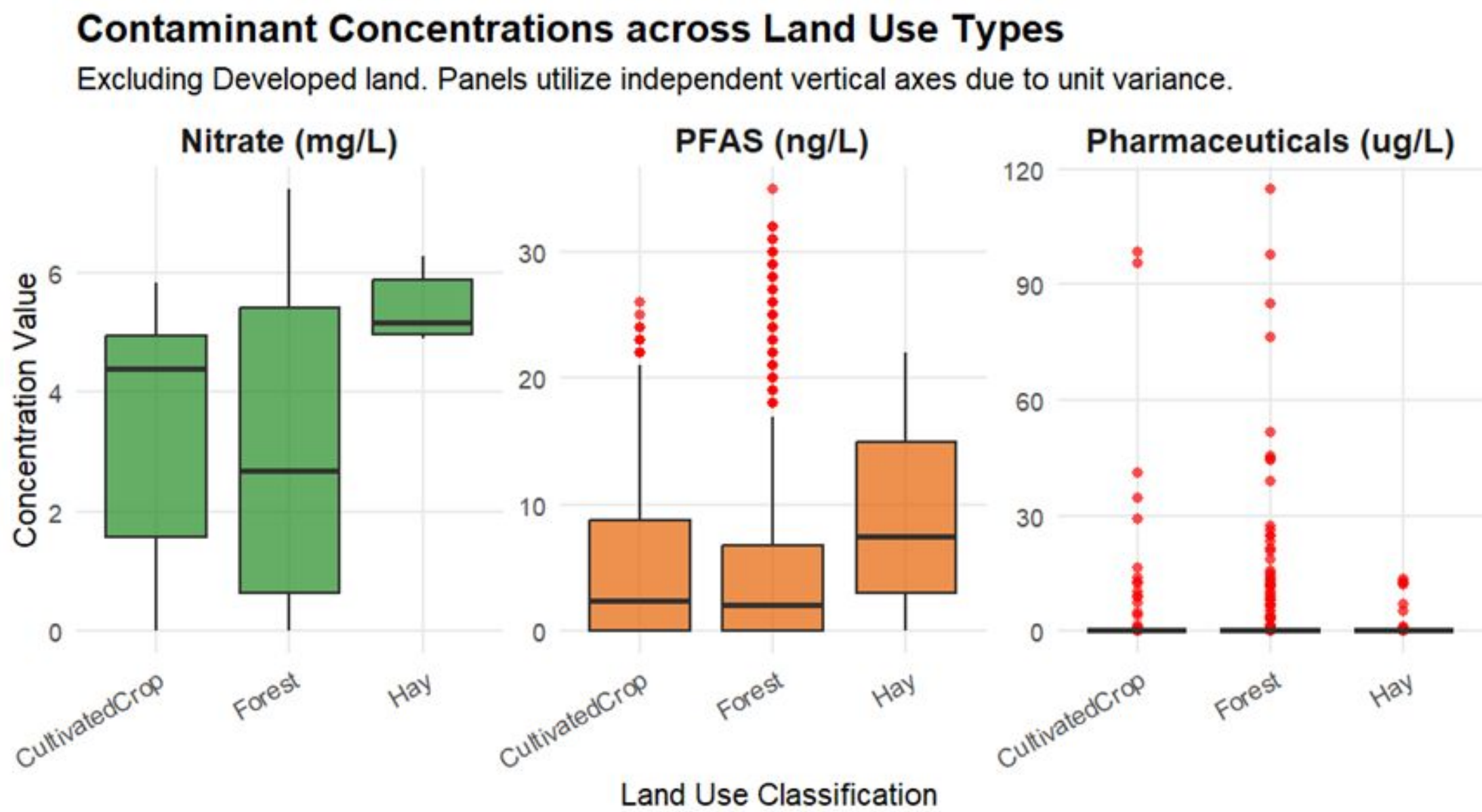

Supplemental Figure S6. Concentration boxplots of nitrate (mg-N/L), PFAS (ng/L), and pharmaceuticals (μg/L) in groundwater underlaying different land use classes.

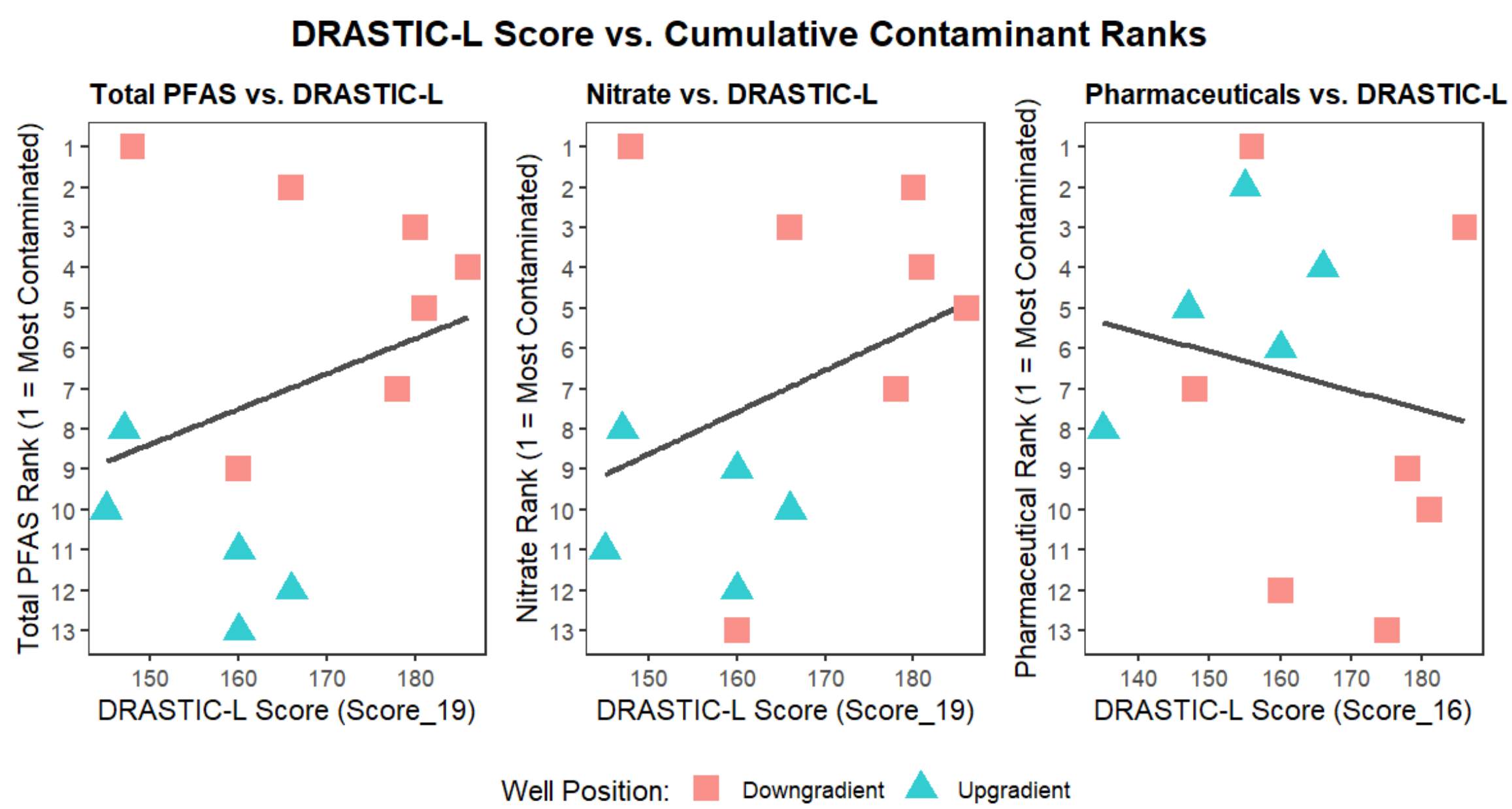

Supplemental Figure S7. Regression plots of DRASTIC-L score and cumulative contaminant rank for total PFAS, nitrate, and pharmaceuticals.

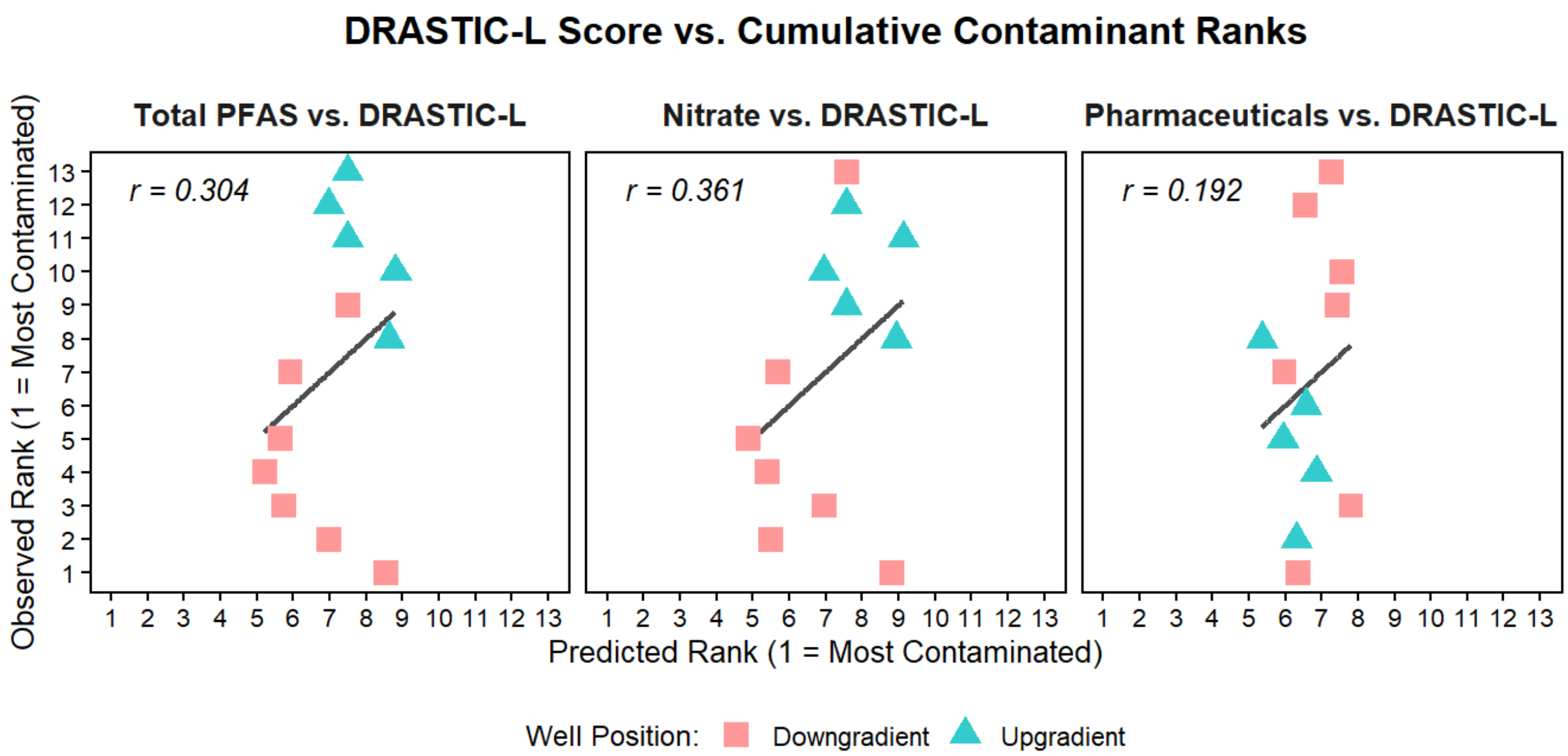

Supplemental Figure S8. Observed versus predicted cumulative contaminant rankings from using DRASTIC-L as the only predictor for total PFAS, nitrate, and pharmaceuticals. Correlation strength (r) between observed versus predicted rank is given.

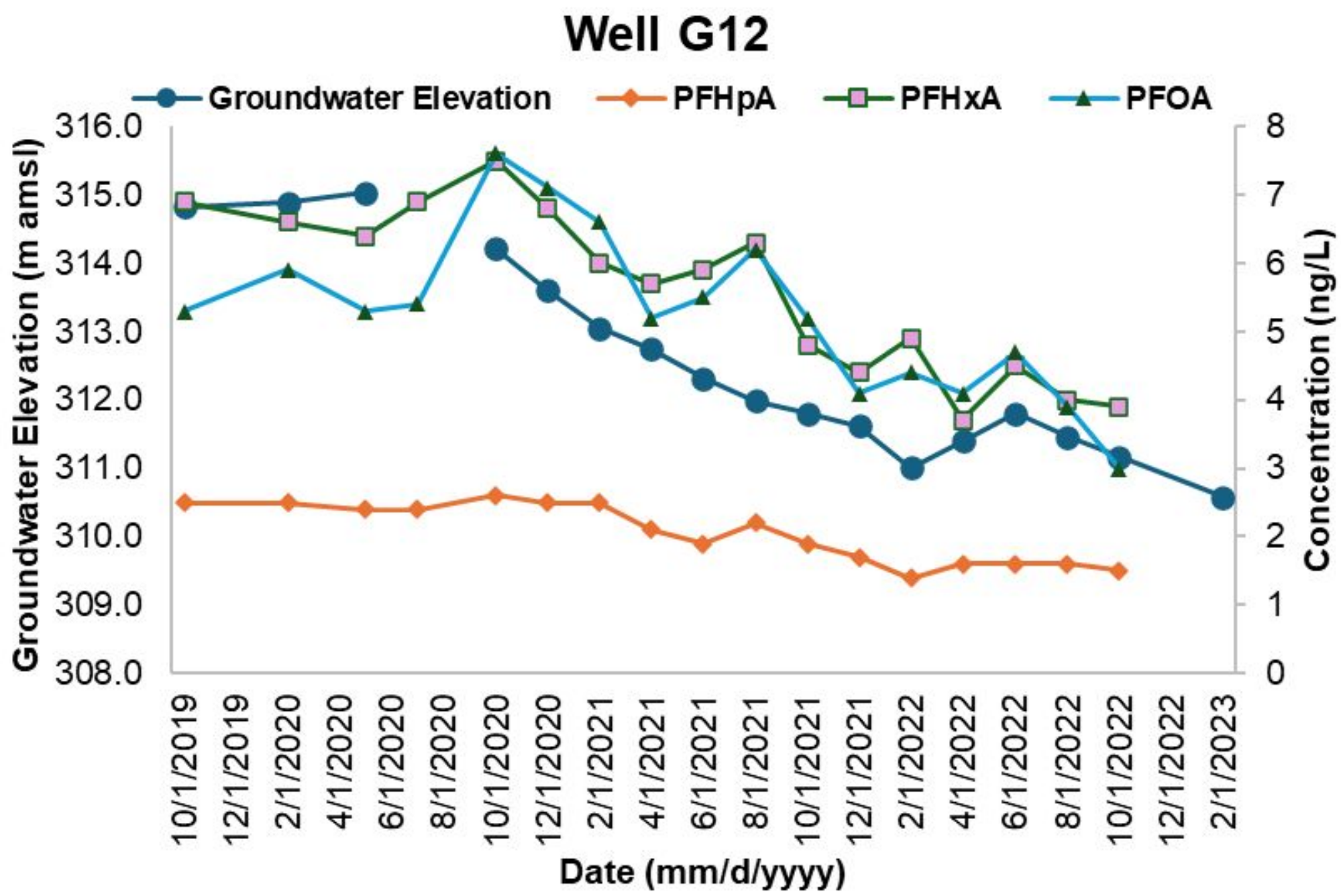

Supplemental Figure S9. Groundwater elevation time series in meters above mean sea level (m asml) and PFAS concentration (ng/L) in well G12.

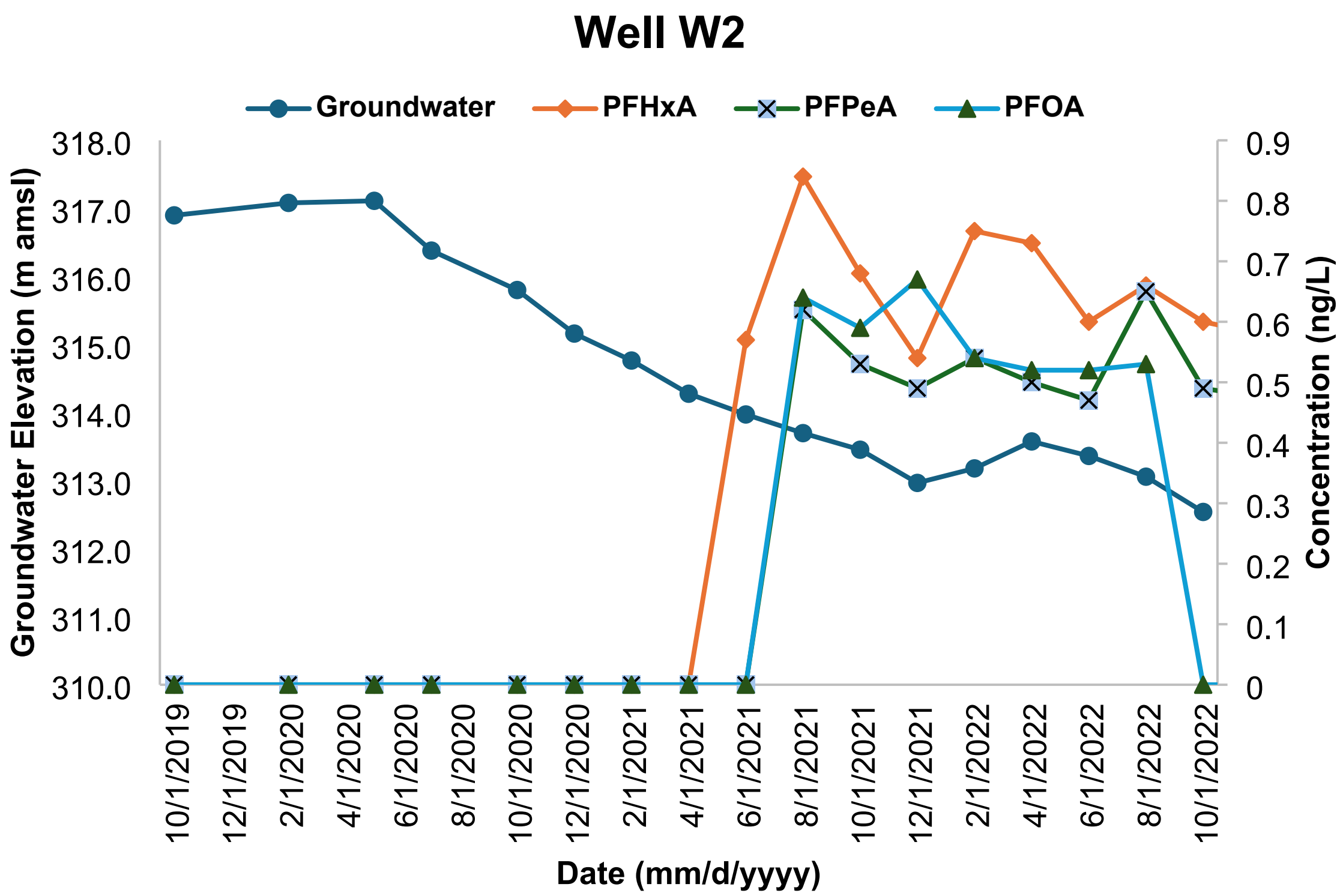

Supplemental Figure S10. Groundwater elevation time series in meters above mean sea level (m asml) and PFAS concentration (ng/L) in well W2.

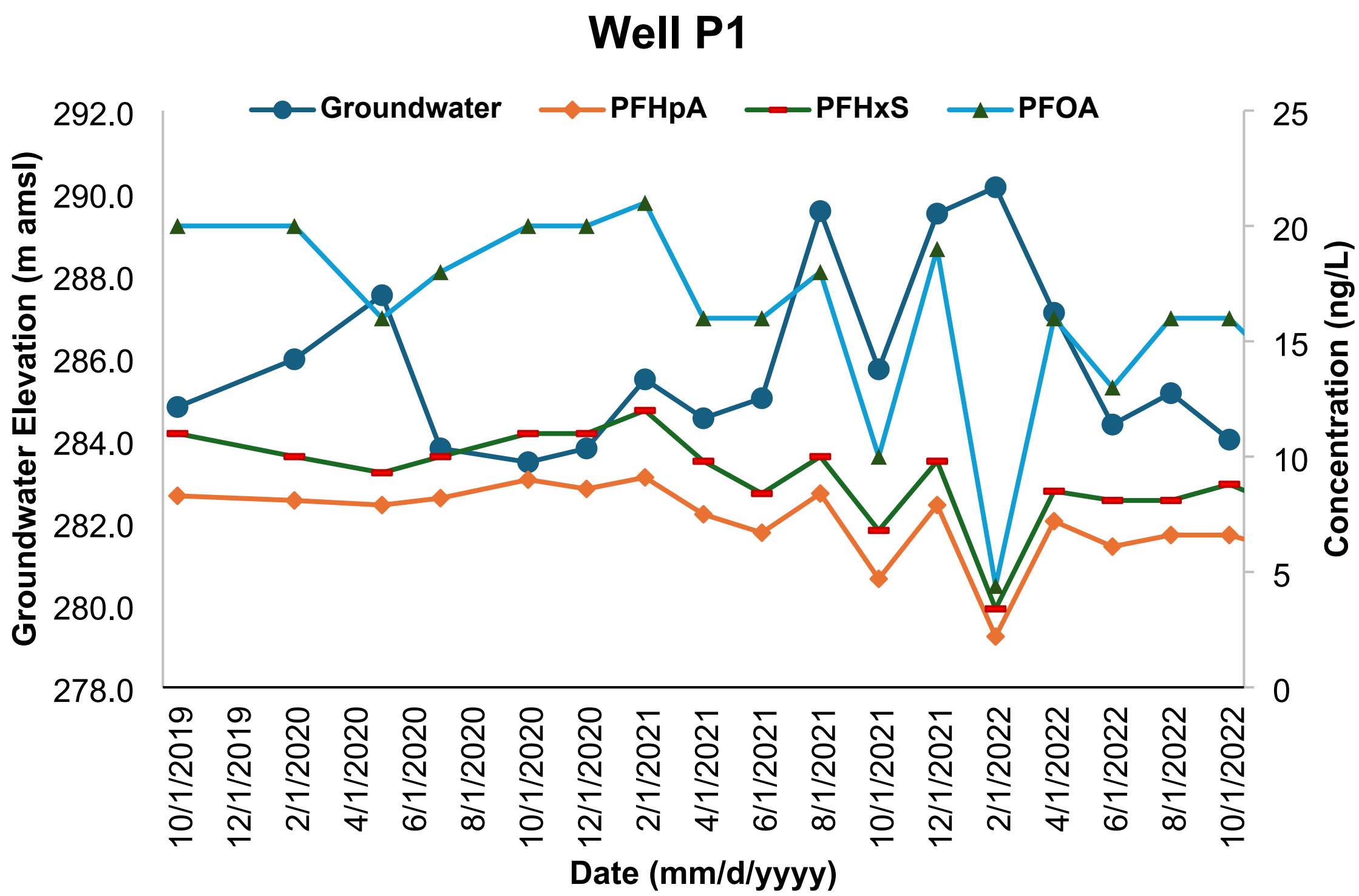

Supplemental Figure S11. Groundwater elevation time series in meters above mean sea level (m asml) and PFAS concentration (ng/L) in well P1.

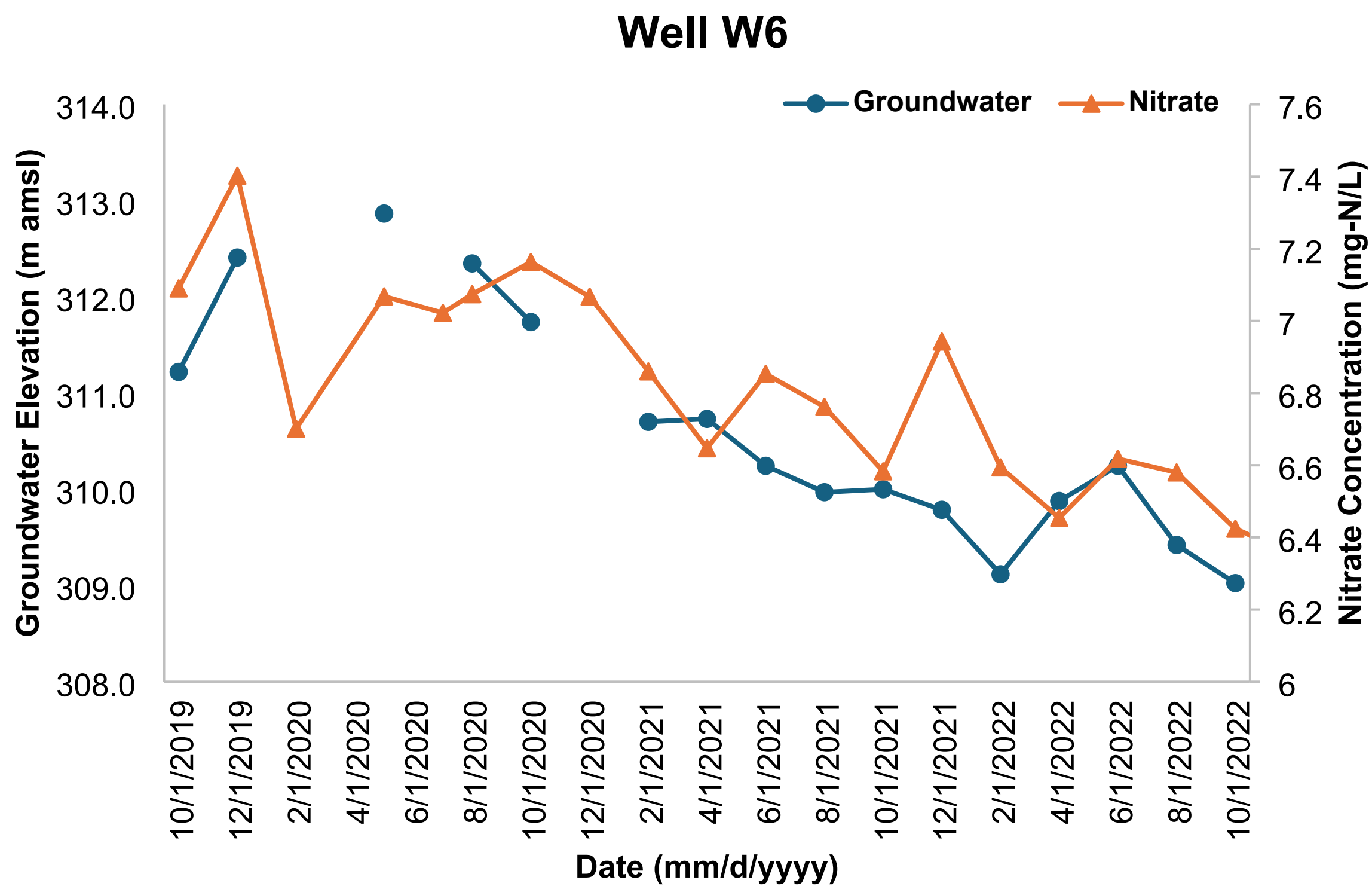

Supplemental Figure S12. Groundwater elevation time series in meters above mean sea level (m asml) and nitrate (mg-N/L) in well W6.

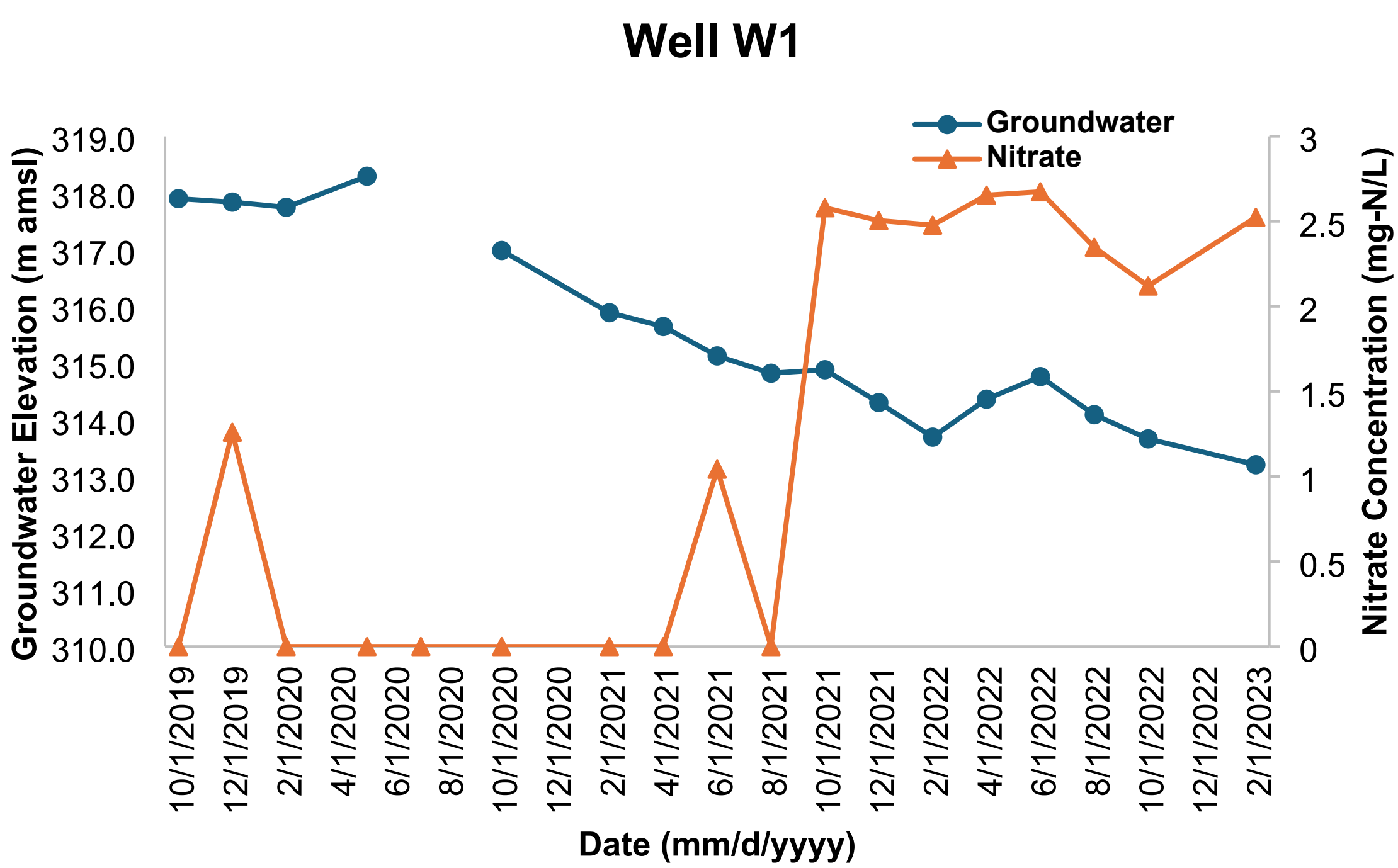

Supplemental Figure S13. Groundwater elevation time series in meters above mean sea level (m asml) and nitrate (mg-N/L) in well W1.

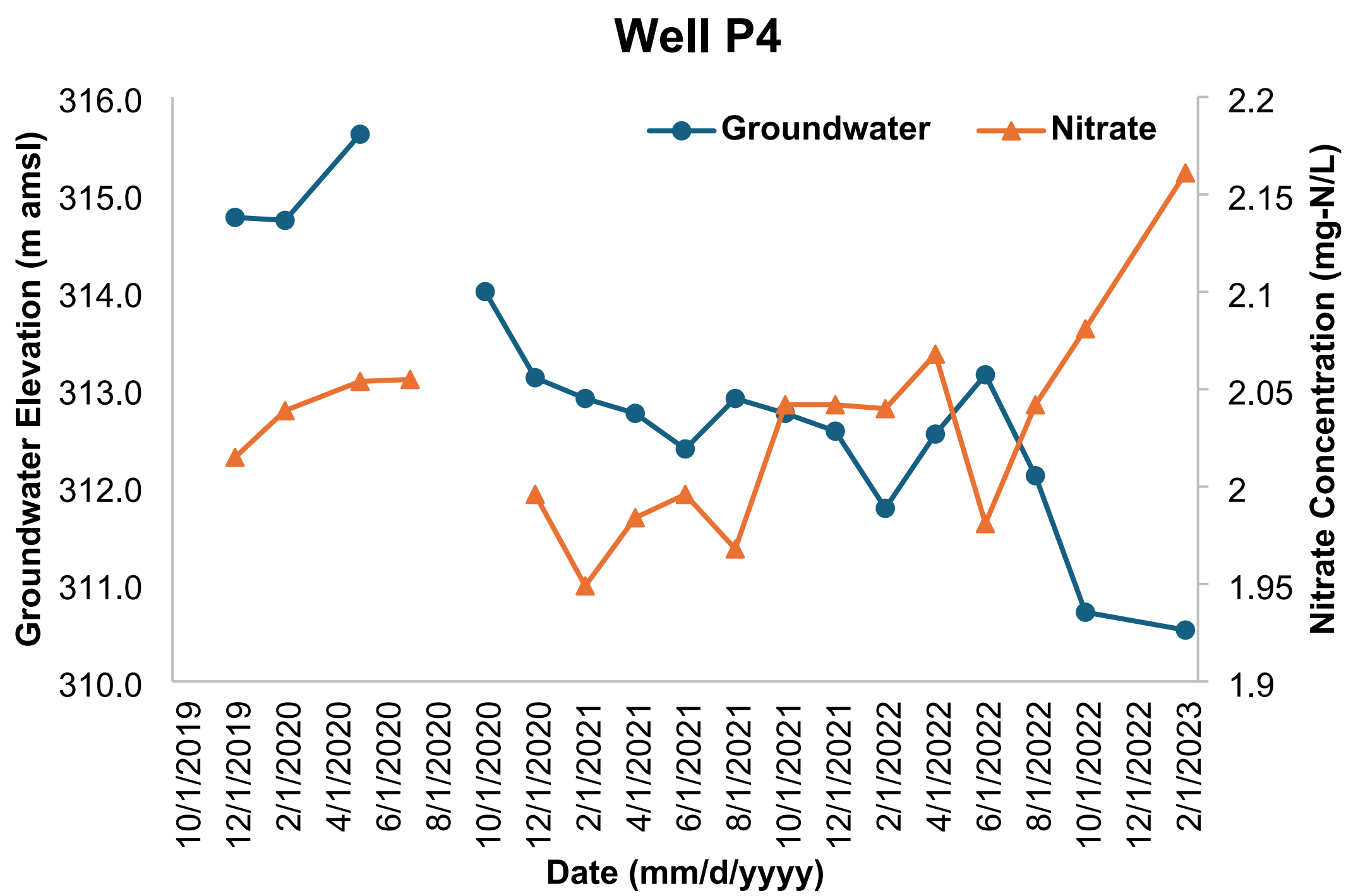

Supplemental Figure S14. Groundwater elevation time series in meters above mean sea level (m asml) and nitrate (mg-N/L) in well P4.

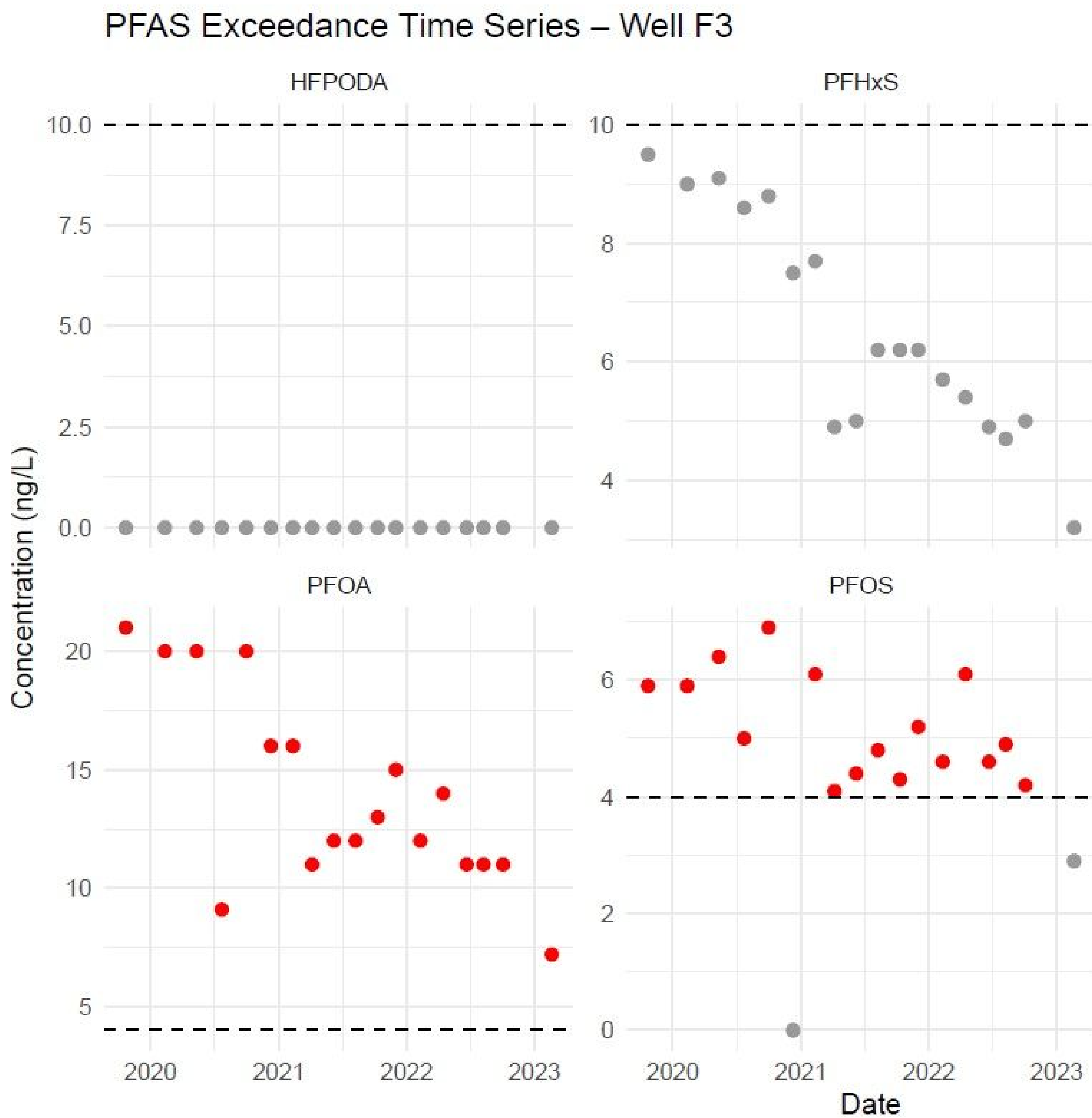

Supplemental Figure S15. PFAS concentration time series compared to the maximum contaminant levels (MCLs) for well F3.

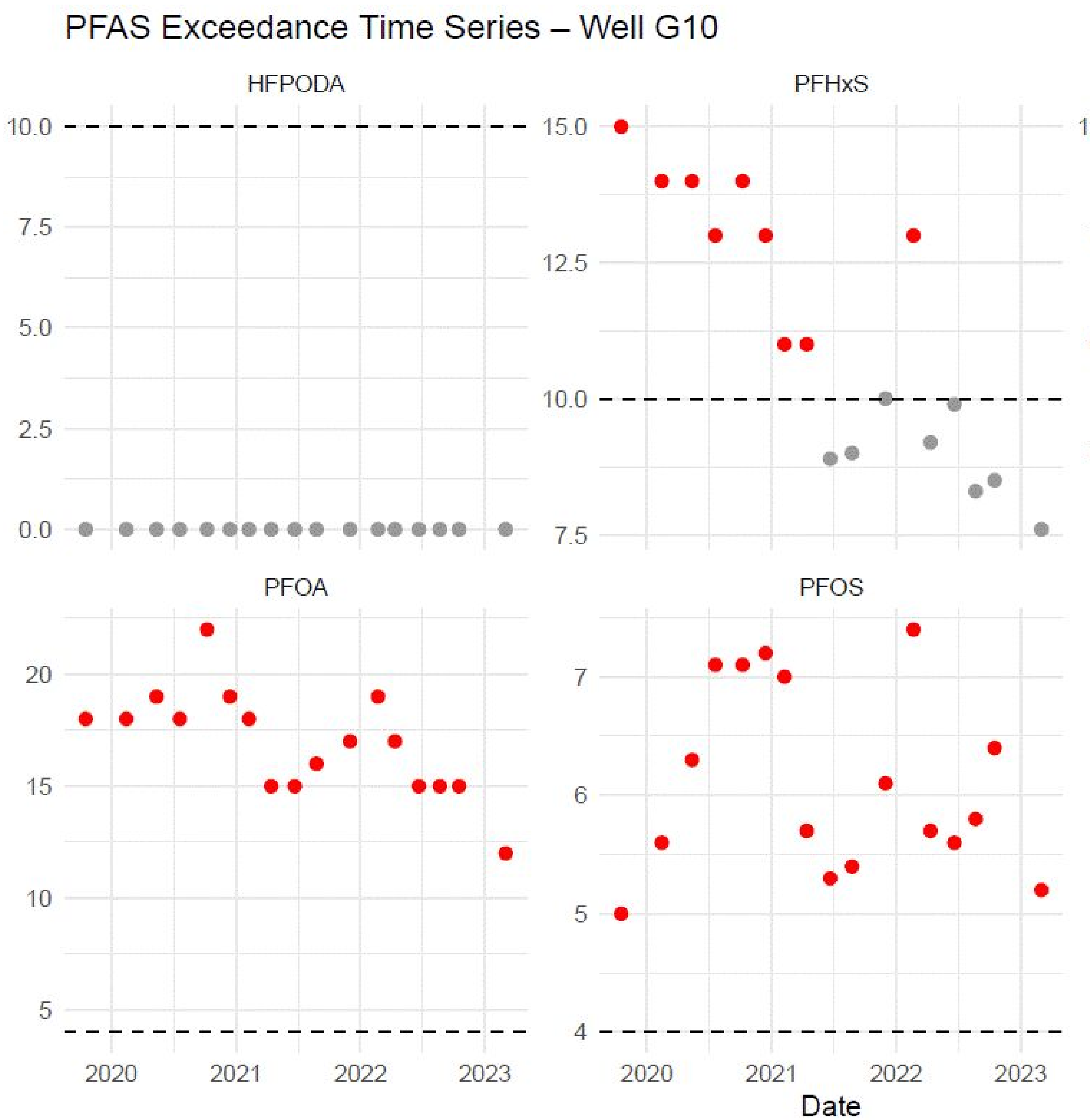

Supplemental Figure S16. PFAS concentration time series compared to the maximum contaminant levels (MCLs) for well G10.

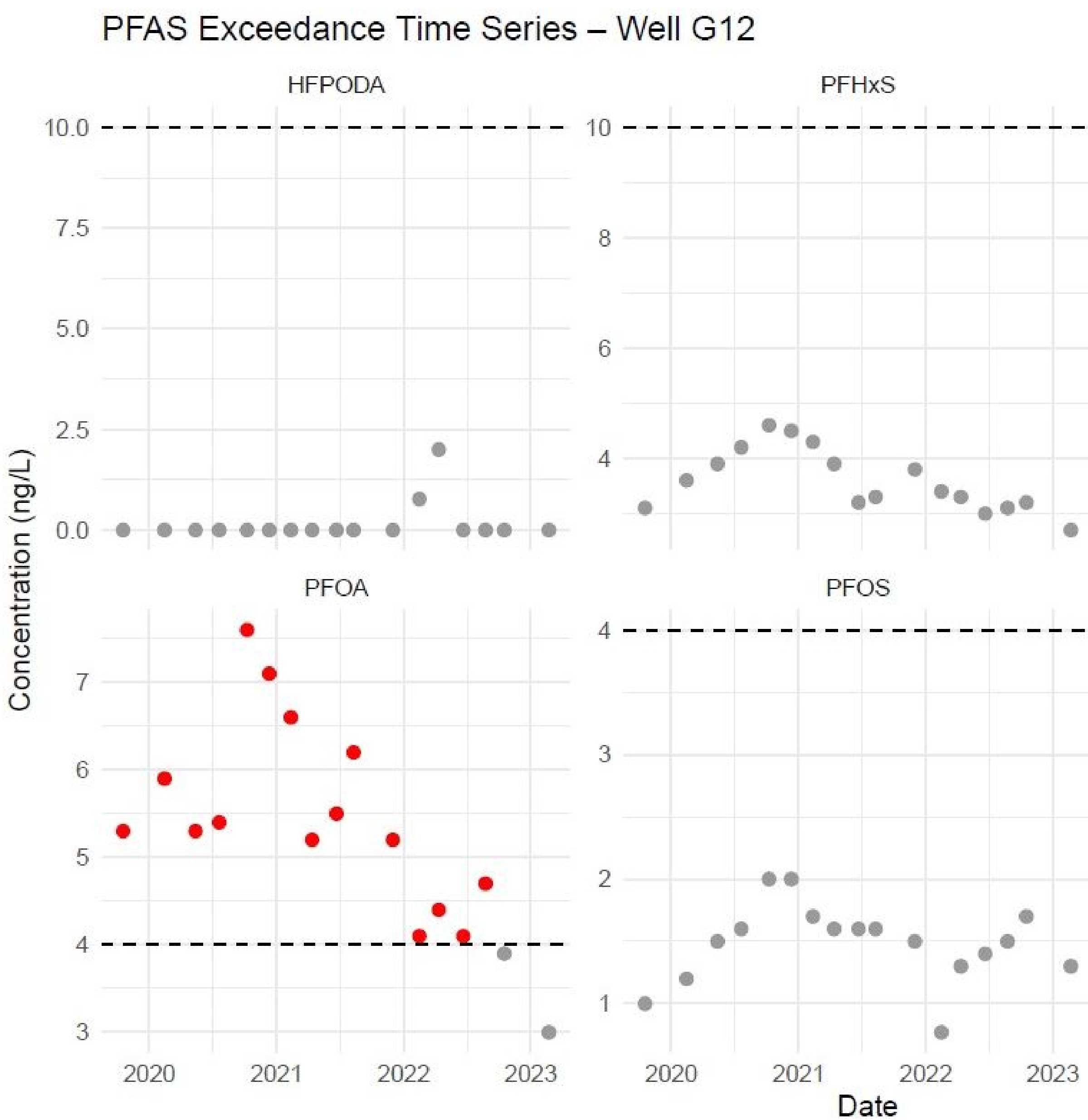

Supplemental Figure S17. PFAS concentration time series compared to the maximum contaminant levels (MCLs) for well G12.

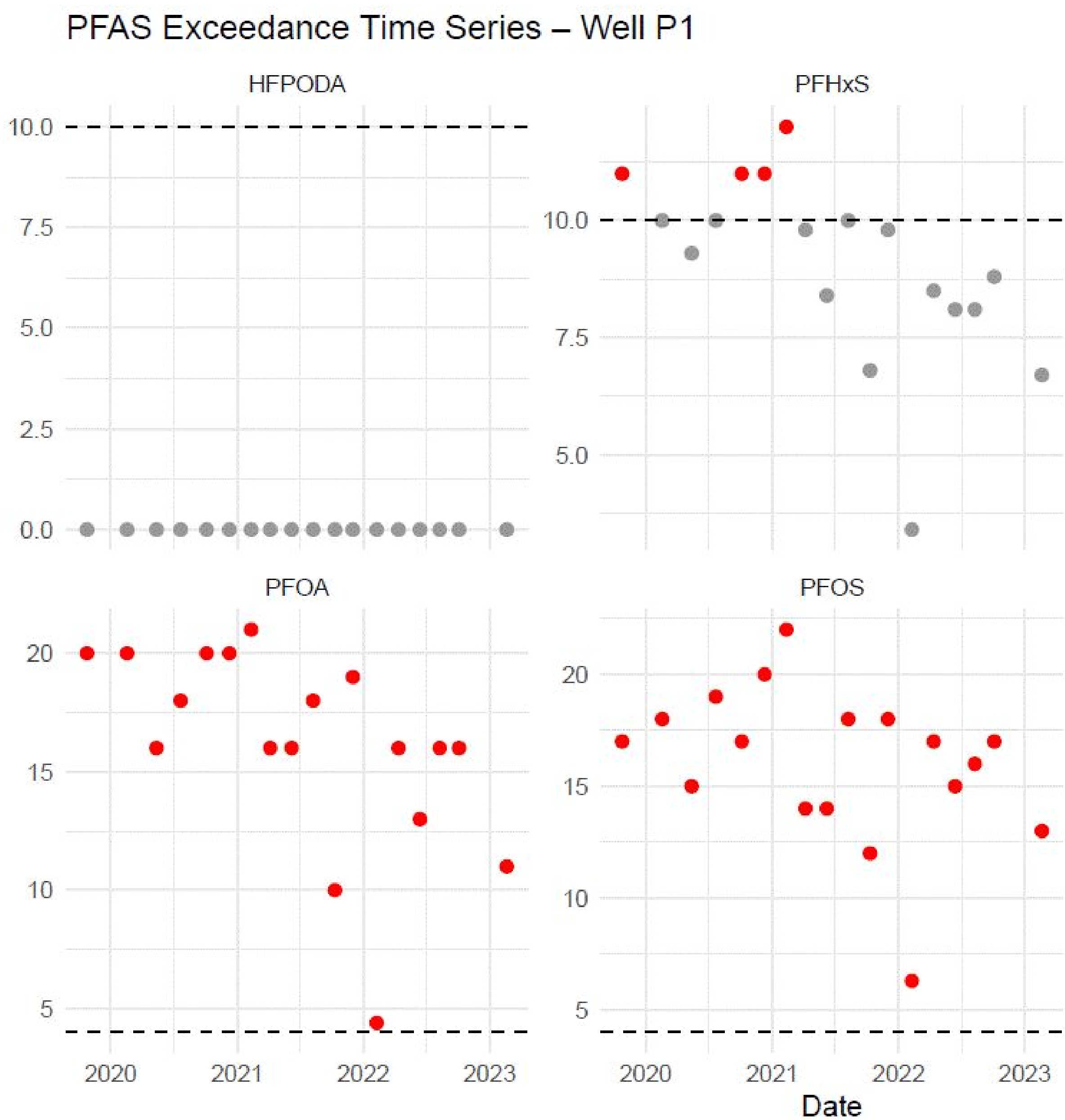

Supplemental Figure S18. PFAS concentration time series compared to the maximum contaminant levels (MCLs) for well P1.

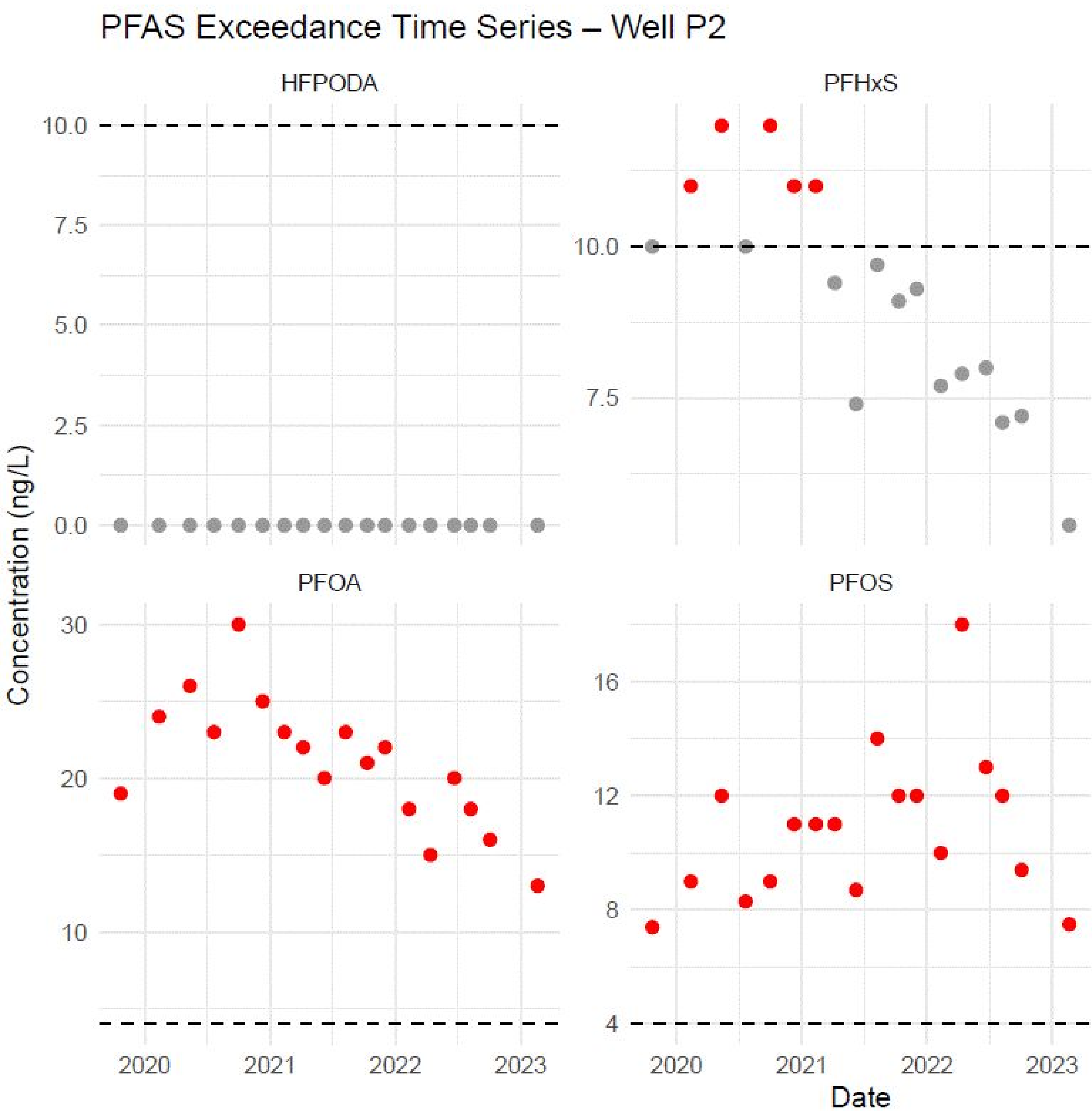

Supplemental Figure S19. PFAS concentration time series compared to the maximum contaminant levels (MCLs) for well P2.

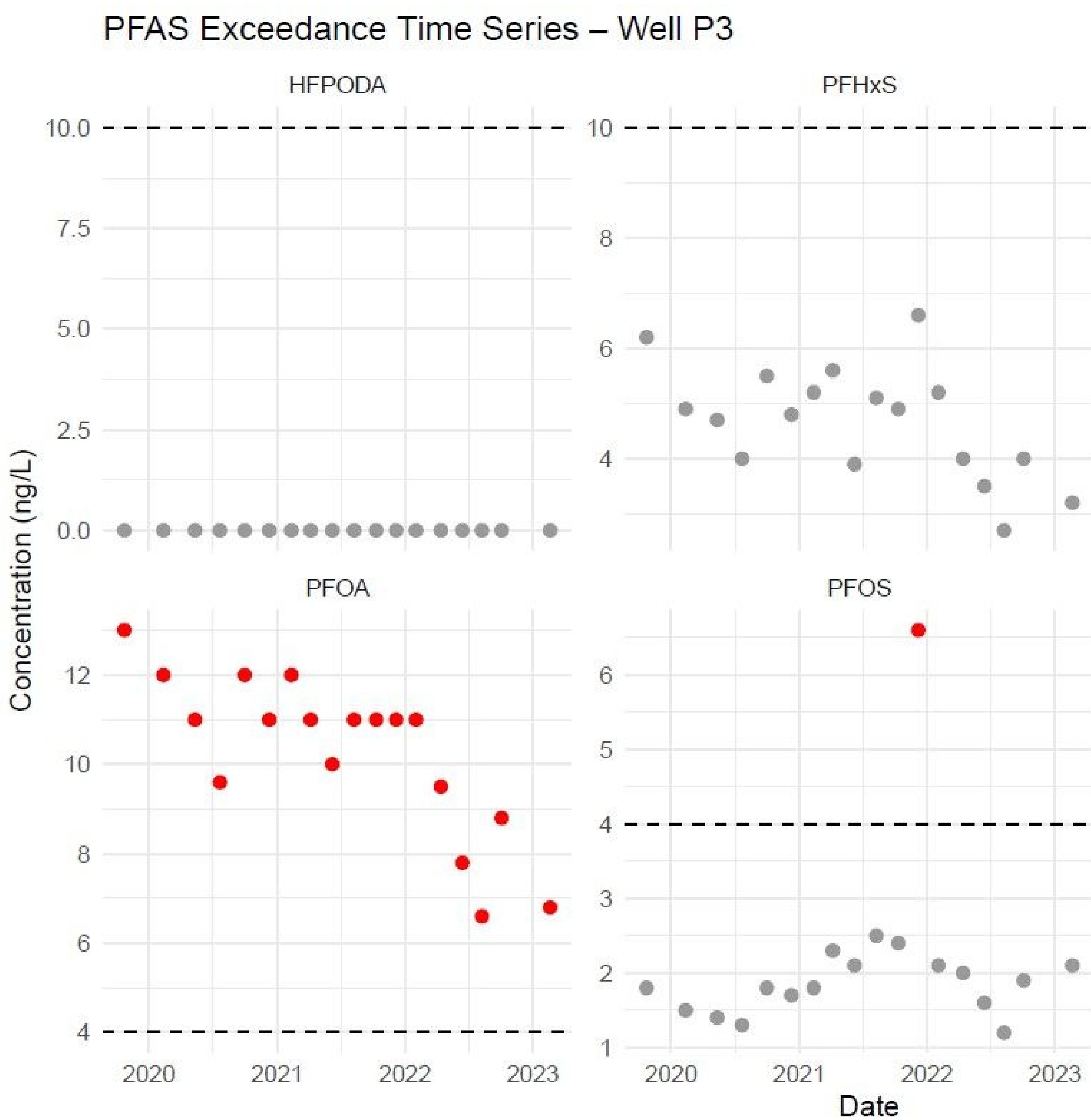

Supplemental Figure S20. PFAS concentration time series compared to the maximum contaminant levels (MCLs) for well P3.

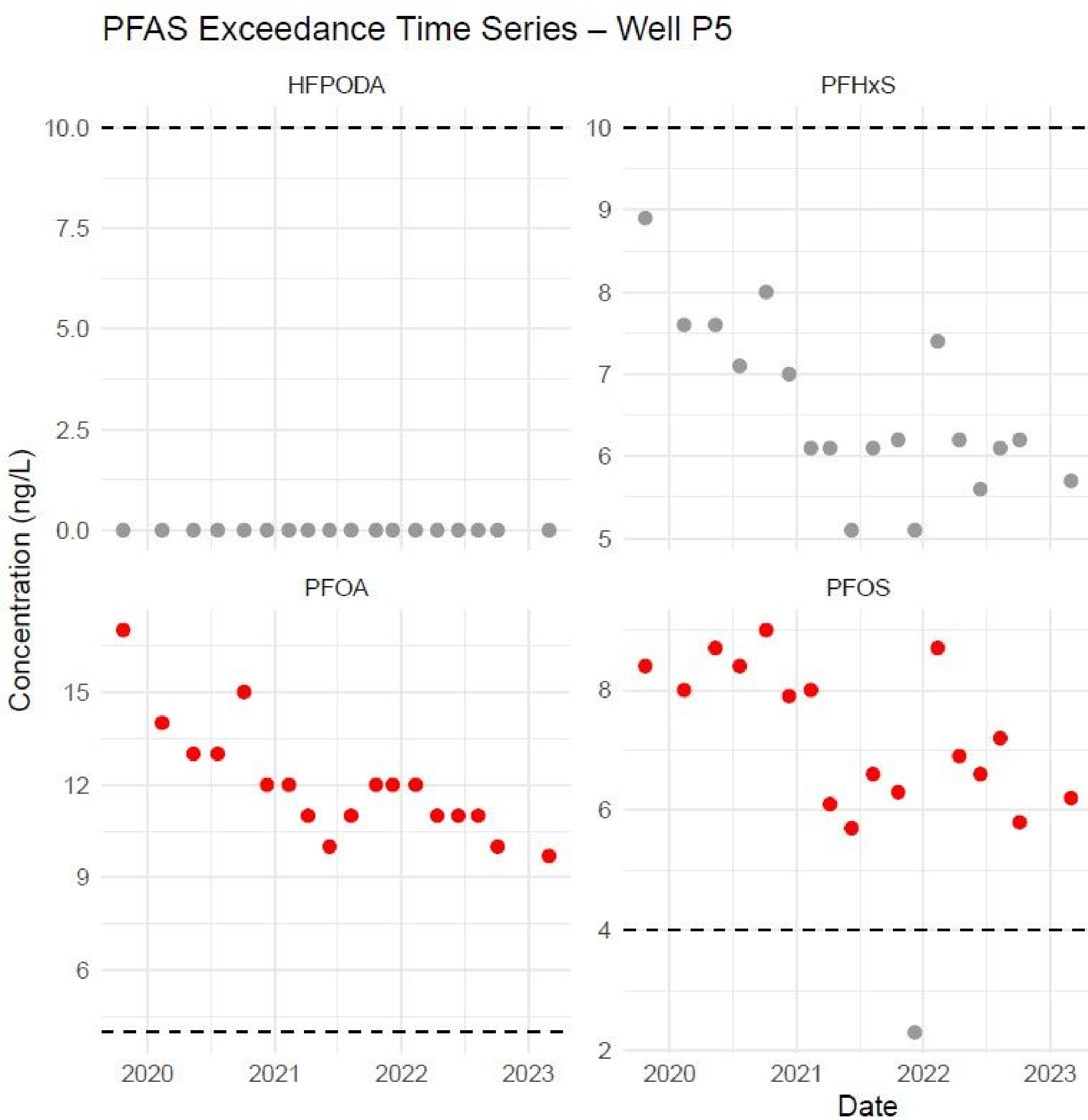

Supplemental Figure S21. PFAS concentration time series compared to the maximum contaminant levels (MCLs) for well P5.

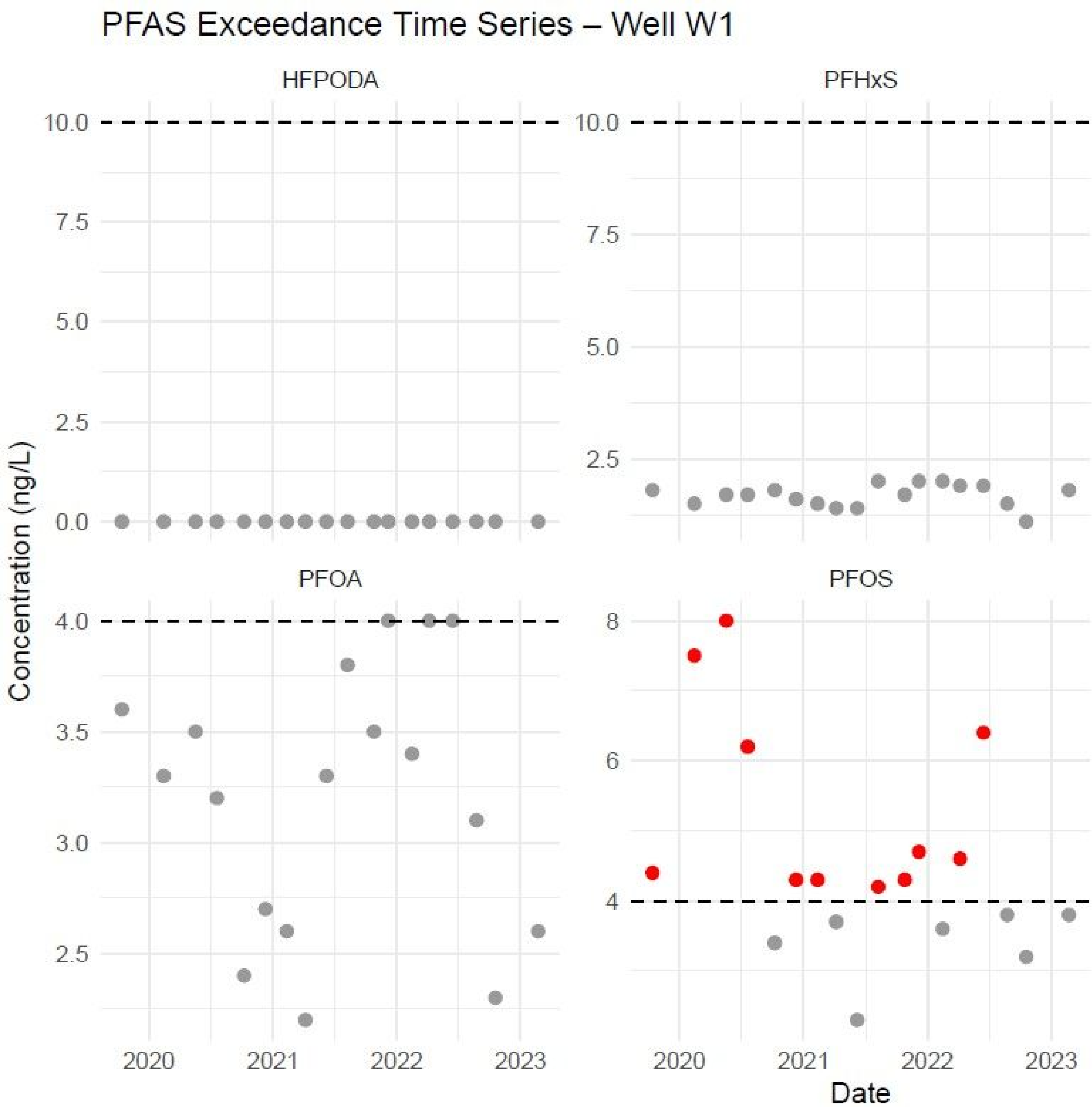

Supplemental Figure S22. PFAS concentration time series compared to the maximum contaminant levels (MCLs) for well W1.

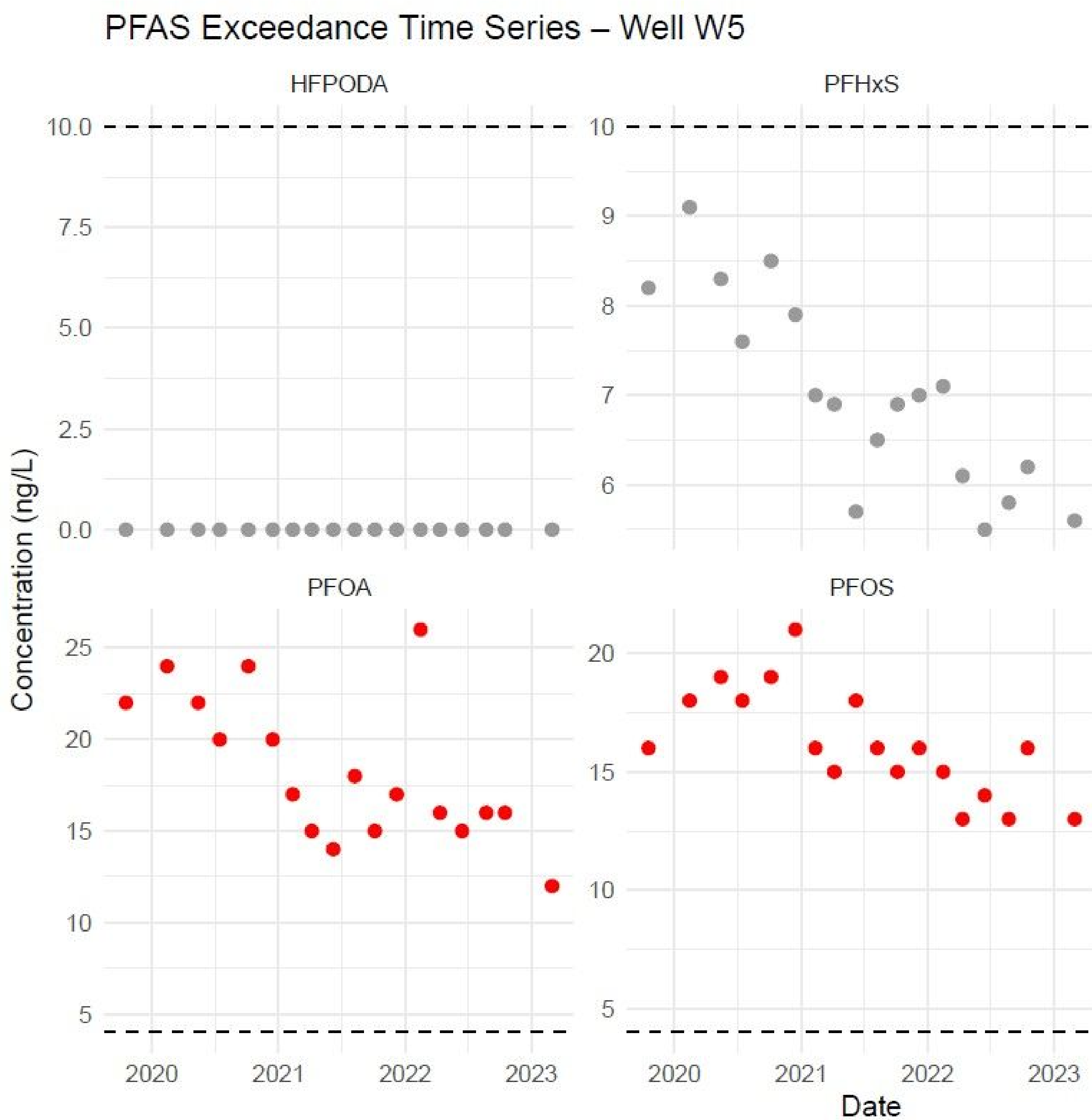

Supplemental Figure S23. PFAS concentration time series compared to the maximum contaminant levels (MCLs) for well W5.

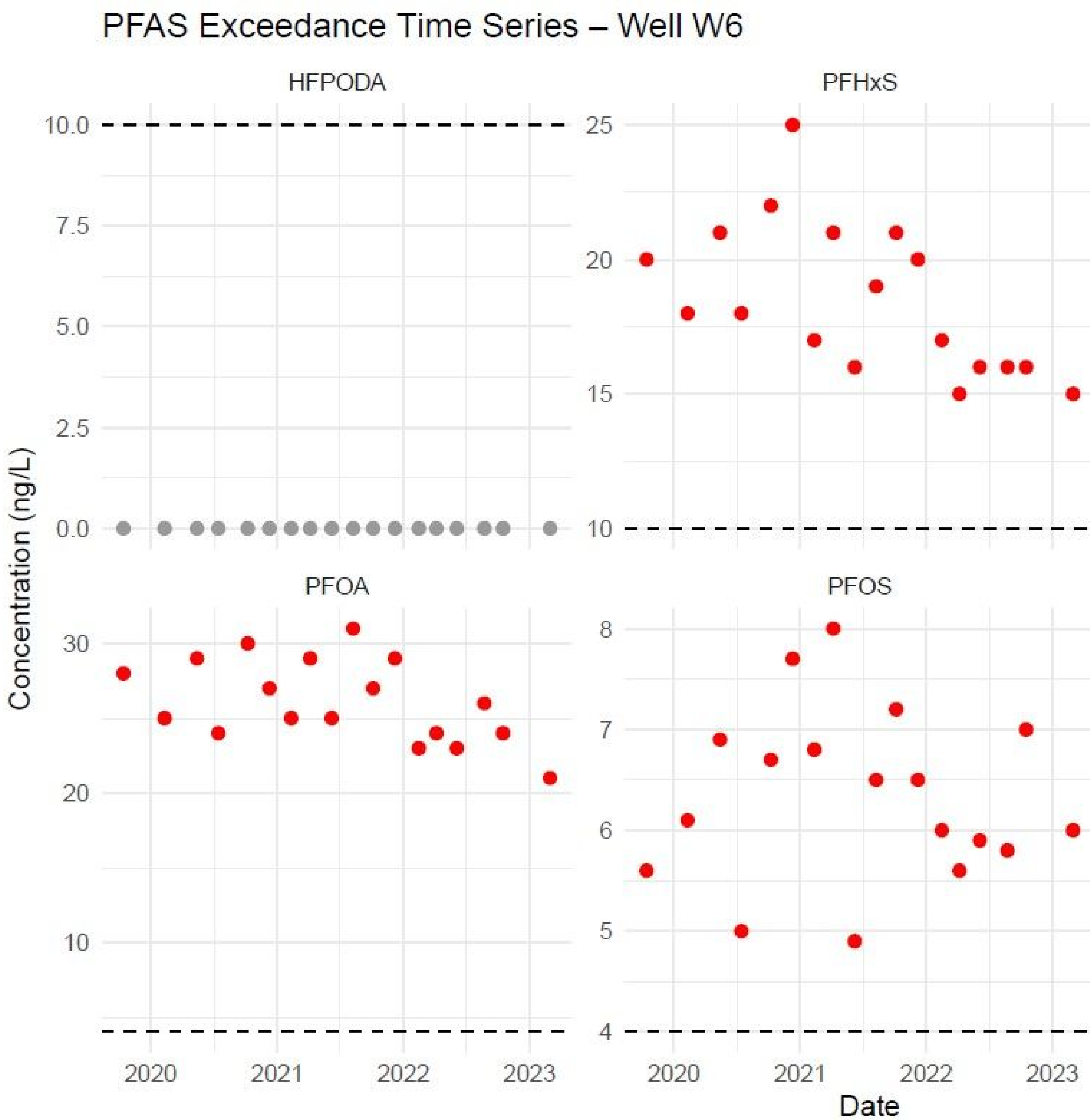

Supplemental Figure S24. PFAS concentration time series compared to the maximum contaminant levels (MCLs) for well W6.

References

1. Kibuye, F. A., Gall, H. E., Elkin, K. R., Ayers, B., Veith, T. L., Miller, M., Jacob, S., Hayden, K. R., Watson, J. E., Elliott, H. A. (2019). Fate of Pharmaceuticals in a Spray-irrigation System: From Wastewater to Groundwater. Sci Tot Environ., 654, 197-208. https://doi.org/10.1016/j.scitotenv.2018.10.442

2. Mroczko, O., Preisendanz, H., Wilson, C., Mashtare, M., Elliott, H., Veith, T., Soder, K., Watson, J. (2022). Spatiotemporal Patterns of PFAS in Water and Crop Tissue at a Beneficial Wastewater Reuse Site in Central Pennsylvania. J Environ Qual, 51(6), 1282-1297. https://doi.org/10.1002/jeq2.20408

3. Shoemaker, J., Tettenhorst, D. (2018). Method 537.1: Determination of Selected Per- and Polyfluorinated Alkyl Substances in Drinking Water by Solid Phase Extraction and Liquid Chromatography/Tandem Mass Spectrometry (LC/MS/MS). U.S. Environmental Protection Agency, Office of Research and Development, National Center for Environmental Assessment, Washington, DC. https://cfpub.epa.gov/si/si_public_record_Report.cfm?dirEntryId=343042&Lab=NERL (accessed 2026-03-19)

4. Menne, M. J., Durre, I., Korzeniewski, B., McNeill, S., Thomas, K., Yin, X., Anthony, S., Ray, R., Vose, R. S., Gleason, B. E., Houston, T. G. (2012a). Global Historical Climatology Network – Daily (GHCN-Daily), Version 3. [Daily Precipitation data for State College, PA (USC00368449), 2016-2023]. NOAA National Climatic Data Center. https://doi.org/10.7289/V5D21VHZ (accessed 2025-09-08)

5. Menne, M. J., Durre, I., Vose, R. S., Gleason, B. E., Houston, T. G. (2012b). An Overview of the Global Historical Climatology Network-Daily Database. J Atmos Oceanic Technol., 29, 897-910. https://doi.org//10.1175/JTECH-D-11-00103.1

6. Multi-Resolution Land Characteristics Consortium. (2021). National Land Cover Database (NLCD) 2021. U.S. Geological Survey. https://www.mrlc.gov/ (accessed 2025-05-30)

7. Pennsylvania Department of Conservation and Natural Resources (PA DCNR). (2007). Digital Data Set of Mapped Karst Features in South-Central and Southeastern Pennsylvania [Data set]. Pennsylvania Spatial Data Access. https://www.pasda.psu.edu/uci/DataSummary.aspx?dataset=3073 (accessed 2025-04-15)

8. Owens, A. (2023). Soil and Landscape Assessment of the Hydrological Capacity of a ~45-Year-Old Spray Irrigation Wastewater Treatment System. Master's Thesis, The Pennsylvania State University. https://etda.libraries.psu.edu/catalog/30883apo5249 (accessed 2025-06-25)

9. Wolock, D. M. Estimated Mean Annual Natural Ground-Water Recharge in The Conterminous United States. (2003). United States Geological Survey (USGS), Open-File Report 2003-311. https://doi.org/10.3133/ofr03311

10. United States Department of Agriculture - Natural Resources Conservation Service (USDA-NRCS) Web Soil Survey. https://websoilsurvey.nrcs.usda.gov/app/ (accessed 2025-09-12)

11. Water, Sanitation, Hygiene, and Health (WSH) team. (2024). Guidance on Wastewater and Solid Waste Management for Manufacturing of Antibiotics. Geneva: World Health Organization (WHO), ISBN: 978-92-4-009725-4. https://www.who.int/publications/i/item/9789240097254 (accessed 2025-08-05)

12. Tsegay, T., Birhanu, B., Azagegn, T., Hailu, B. T., Ayenew, T. (2024). Assessing Groundwater Vulnerability to Pollution in a Rapidly Urbanizing River Basin Using a Modified DRASTIC Land Use-Lineament Density Method. Geol J, 59(12), 3278-3295. https://doi.org/10.1002/gj.5059
